# Improved efficacy and long‐term protective effects of CXCR4/IL10 bioengineered mesenchymal stromal cells in a model of inflammatory bowel disease

**DOI:** 10.1002/btm2.70083

**Published:** 2025-12-16

**Authors:** Mercedes Lopez‐Santalla, Marta C. Ordoñez‐Velasco, Maria Fernandez‐Garcia, Miriam Hernando‐Rodriguez, Juan A. Bueren, Rosa M. Yañez, Marina I. Garin

**Affiliations:** ^1^ Division of Hematopoietic Innovative Therapies Centro de Investigaciones Energéticas, Medioambientales y Tecnológicas (CIEMAT) and Centro de Investigación Biomédica en Red de Enfermedades Raras (CIBER‐ER) Madrid Spain; ^2^ Advanced Therapy Unit Instituto de Investigación Sanitaria Fundación Jiménez Díaz (IIS‐FJD/UAM) Madrid Spain; ^3^ Kiji Therapeutics Paris France

**Keywords:** CXCR4, IL10, immunomodulation, inflammatory bowel disease, mesenchymal stromal cell‐based therapy

## Abstract

Mesenchymal stromal cell (MSCs)‐based therapies have emerged as a promising approach for inflammatory bowel disease (IBD) treatment due to their immunosuppressive and regenerative properties. However, clinical trials have shown limited therapeutic effectiveness, largely because of low efficiency in penetrating the inflamed colon and their inconsistent in vivo immunomodulatory ability. In this study, we generated genetically engineered adipose‐derived human MSCs constitutively expressing CXC chemokine receptor 4 and interleukin 10 (CXCR4‐IL10‐MSCs) to promote their delivery to the inflamed colon and enhance their immunosuppressive capability. Compared to unmodified MSCs, CXCR4‐IL10‐MSCs exhibited enhanced trafficking to the inflamed colon and achieved improved therapeutic effects in dextran sulfate sodium (DSS)‐challenged colitic mice. Upon a chronic DSS re‐challenge, CXCR4‐IL10‐MSCs showed enhanced long‐term protective effects. These findings demonstrate that stable ectopic expression of CXCR4 and IL10 enhances the therapeutic efficacy of MSCs and supports the development of an optimized MSC‐based product capable of inducing an improved long‐term protective immune memory in IBD.

**Abbreviations**
Ad‐MSCs: adipose‐derived mesenchymal stromal cells
Arg‐1: arginase‐1
CMs: classical monocytes
COX: cyclooxygenase
CXCR: C‐X‐C chemokine receptor type
DAI: disease activity index
DSS: dextran sulfate sodium
FOXP3: Forkhead box P3
GM‐CSF: granulocyte‐macrophage colony‐stimulating factor
GMP: granulocyte–monocyte progenitors
IBD: inflammatory bowel disease
IFN: interferon
IL: interleukin
IMIDs: immune‐mediated inflammatory diseases
iNOS: inducible nitric oxide synthase
IP: intraperitoneal
IV: intravenous
LPS: lipopolysaccharide
MDP: monocyte‐dendritic cell progenitors
MHC: major histocompatibility complex
NCMs: non‐classical monocytes
PB: peripheral blood
PBS: phosphate‐buffered saline
PDL‐1: programmed cell death 1 ligand
RAG: recombination‐activating gene
ROI: regions of interests
SDF‐1: stromal cell‐derived factor‐1
TAPI‐1: TNF alpha protease inhibitor I
TGF: transforming growth factor
TNBS: 2,4,6‐trinitrobenzenesulfonic acid
TNF: tumor necrosis factor
WT: wild type

**Translational Impact Statement**

This study highlights the therapeutic potential of CXCR4‐IL10 dual‐engineered mesenchymal stromal cells (MSCs) as an advanced cell‐based therapy for inflammatory bowel disease (IBD), offering improved targeting of inflamed tissue and enhanced immunomodulation. These findings support further development towards clinical application, with the goal of achieving more effective and durable treatment outcomes for patients with IBD.

## 1 INTRODUCTION

Mesenchymal stromal cell (MSCs) therapy has emerged as a promising strategy for treating immune‐mediated inflammatory diseases (IMIDs) such as rheumatoid arthritis^1^ and inflammatory bowel disease (IBD).^2^ These conditions present a significant societal and economic burden due to their chronic nature, high treatment costs, and associated morbidity. Current therapies, including corticosteroids, immunosuppressants, and biologics, often fail to provide long‐term effects, with many patients developing resistance, experiencing severe side effects, or becoming refractory over time.^3^, ^4^ MSCs are of particular interest due to their potent immunomodulatory properties, which include interactions with T cells, modulation of macrophages, and secretion of anti‐inflammatory cytokines, as well as their ability to promote tissue regeneration.^5^, ^6^ To date, nearly a 1000 clinical trials have investigated MSC‐based therapies, with approximately 100 specifically targeting IMIDs.^5^, ^7^, ^8^ While MSCs have demonstrated efficacy in preclinical studies, their inconsistent effectiveness in clinical trials remains a major obstacle to clinical application.^9^ To address these challenges, the scientific and clinical communities are exploring strategies to enhance MSC therapeutic potency, which focus on patient targeting and stratification, defining quality attributes of MSCs, and developing next‐generation MSC products.

Building on our previous findings, where MSCs engineered to express C‐X‐C chemokine receptor type 4 (CXCR4) and interleukin (IL) 10 (CXCR4‐IL10‐MSCs) demonstrated enhanced therapeutic effects in a humanized model of graft‐versus‐host disease^10^ and in a lipopolysaccharide (LPS)‐induced inflamed pad model,^11^ in this study we aimed to evaluate the therapeutic efficacy of CXCR4‐IL10‐MSCs in a preclinical model of IBD.

IBD is characterized by inflammation of the intestinal mucosa of the digestive tract, with increasing global prevalence attributed to factors such as industrialization, diet, and genetic predisposition. While its precise etiology remains unclear, key contributors include immune dysregulation, epithelial barrier dysfunction, and microbiota dysbiosis.^12^ Stromal cell‐derived factor 1 (SDF‐1/CXCL12), the ligand for CXCR4, enhances MSC migration towards inflamed tissues^13^, ^14^ including the colon, as observed in DSS‐induced colitis mouse models.^15^, ^16^ Significant upregulation of SDF‐1 has been documented in the colon of IBD patients.^17^ Additionally, IL10, an anti‐inflammatory cytokine essential for gastrointestinal homeostasis,^18^ has been shown to alleviate IBD‐like symptoms in both preclinical^19^ and clinical settings.^20^, ^21^ However, systemic administration of IL10 has been hindered by suboptimal cytokine delivery to inflamed tissues^22^ and dose‐limiting side effects such as anemia and thrombocytopenia.^21^, ^23^ To address these limitations, advanced delivery methods for IL10, like nanoparticles and fusion proteins, have been used in arthritis,^24^ atherosclerosis,^25^, ^26^ cancer^27^ and IBD, providing sustained release to target inflamed sites while reducing inflammation. In IBD, a phase I study using *Lactococcus lactis* engineered to secrete IL10 demonstrated feasibility and localized delivery but highlighted challenges in achieving consistent mucosal dosing.^28^ A nanoparticle–microsphere system delivering IL10 plasmids improved experimental colitis in rodents, yet faced issues with formulation complexity and stability during gastrointestinal transit.^29^ Gut‐selective oral IL10 fusion proteins also showed promising preclinical activity by crossing the intestinal barrier, though concerns about localized effects with systemic exposure risks.^30^ Collectively, these studies emphasize that while IL10 holds therapeutic promise for IBD, clinical translation requires localized, durable, and well‐controlled delivery strategies.

By taking advantage of the chemotactic axis SDF‐1/CXCR4, our approach aims to achieve the targeted homing of MSCs to inflamed colonic tissue, where localized delivery of IL‐10 would effectively reduce inflammation. This strategy addresses key limitations of systemic IL‐10 administration by ensuring site‐specific cytokine release, thereby maximizing therapeutic efficacy while minimizing off‐target effects.

In this study, we demonstrate that genetically engineered CXCR4‐IL10‐MSCs improve both the short‐term and long‐term therapeutic effects of unmodified MSCs in a DSS‐induced colitic mouse model and demonstrate the potential of this advanced cell‐based therapy for IBD.

## 2 RESULTS

### 2.1 Assessment of CXCR4‐IL10‐MSC‐based therapy in a DSS‐induced colitis mouse model

To evaluate whether CXCR4‐IL10‐MSCs could serve as a potential alternative cell therapy for IBD, we tested the therapeutic efficacy of these cells in a (DSS)‐induced colitis mouse model. As in previous studies,^31^ a single dose of 3 × 10^6^ million WT‐MSCs or CXCR4‐IL10‐MSCs per mouse was administered intraperitoneally on day 5 of the 7‐day DSS cycle.

**FIGURE 1:**
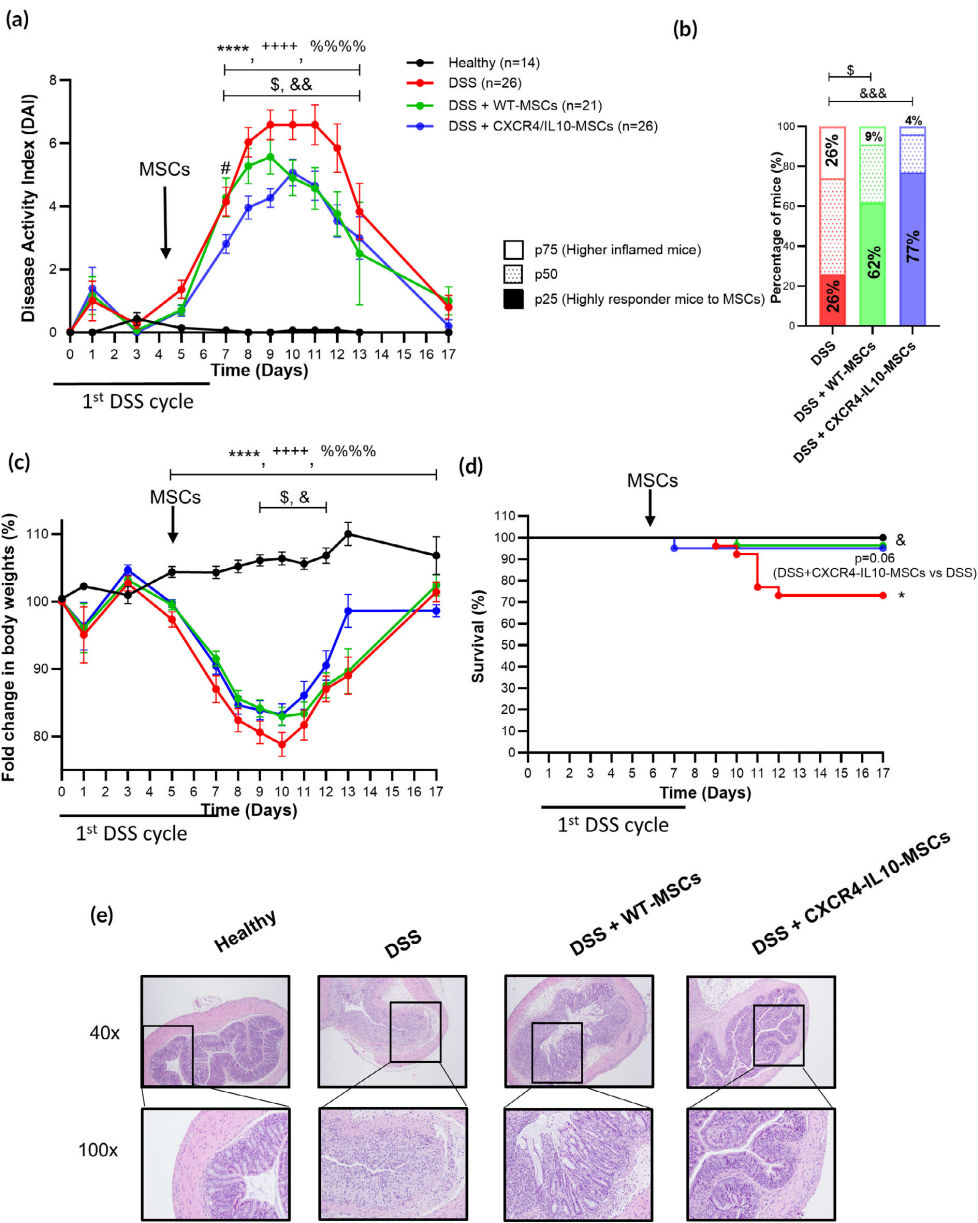
Colitis status of mice following a 7‐day DSS cycle with or without a single intraperitoneal infusion of WT‐ or CXCR4‐IL10‐MSCs. (a) Disease activity index (DAI), (b) contingency graph in percentage of colitic mice classified as WT‐ and CXCR4‐IL10‐MSC‐treated and untreated DSS colitic mice based on percentiles [p25, p50, and p75] of their cumulative DAI, (c) fold change in body weights and (d) survival during the 7‐day DSS cycle. (e) Representative images of colon tissue (magnification 40× and 100×) on day 9 after DSS treatment. Data are presented as mean ± standard error of the mean for DAI and fold change in body weights (percentage of initial body weight). Survival data are shown as percentages. Significance was analyzed using the Mann–Whitney *U* test for DAI and body weight loss, the log rank test for survival, and Fisher's test for the contingency graph. Significant levels are indicated as *p < 0.05 and ****p ≤ 0.0001 DSS versus healthy, ^++++^ *p* < 0.0001 WT‐MSCs versus healthy, ^%%%%^ *p* < 0.0001 CXCR4‐IL10‐MSCs versus healthy, ^$^ *p* < 0.05 DSS + WT‐MSCs versus DSS, ^&^ *p* < 0.05, ^&&^ *p* < 0.01, and ^&&&^ *p* < 0.001 DSS + CXCR4‐IL10‐MSCs versus DSS and ^#^ *p* < 0.05 DSS + WT‐MSCs versus DSS + CXCR4‐IL10‐MSCs. Healthy, *n* = 14; DSS, *n* = 26; DSS + WT‐MSCs, *n* = 26; and DSS + CXCR4‐IL10‐MSCs, *n* = 21. Results represent five independent experiments.

As shown in Figure 1, the 7‐day DSS cycle led to a marked increase in disease activity index (DAI, 6.6 ± 0.0 peak at day 10, Figure 1a), body weight loss (78.8 ± 0.0% maximum body weight loss at day 10, Figure 1c), and decreased survival (73%, Figure 1d) in untreated DSS‐induced colitic mice compared to healthy mice (0.1 ± 0.0, 106.4 ± 0.0% and 100%, respectively). Compared to non‐MSC treated colitic mice, colitic mice treated with WT‐MSCs had significantly reduced body weight loss (83.2 ± 0.0%) and increased survival (96%) along with a reduced DAI at day 9 (5.6 ± 0.0). CXCR4‐IL10‐MSCs significantly enhanced the therapeutic effect of WT‐MSCs, as demonstrated by a delayed and reduced peak of DAI at day 10 (5.1 ± 0.0). Histological analysis on day 9 revealed a more preserved colon morphology and reduced leukocyte infiltration in CXCR4‐IL10‐MSC‐treated colitic mice compared to WT‐MSC‐treated and untreated colitic mice on day 9 (Figure 1e).

To assess individual responses, the cumulative disease activity index was calculated for untreated, WT‐MSC, and CXCR4‐IL10‐MSC‐induced colitic mice during the 7‐day DSS cycle, as we had done in previous studies.^31^ Mice were then stratified into percentiles (p25, p50, and p75) based on their responses. As shown in Figure 1b, a significantly higher number of responder mice was observed in the group treated with CXCR4‐IL10‐MSCs (77%, p25) compared with the WT‐MSC‐treated group (62%) and the untreated colitic group (26%).

These findings show that the stable ectopic expression of CXCR4 and IL10 in Ad‐MSCs improved the therapeutic effect of MSCs in an experimental model of inflammatory bowel disease.

### 2.2 In vivo biodistribution of WT‐MSCs and CXCR4‐IL10‐MSCs

To investigate the differences in the in vivo trafficking of CXCR4‐IL10‐MSCs compared to WT‐Ad‐MSC to different tissues and organs, the biodistribution of both cell types was analyzed in healthy and DSS‐induced colitic mice from 2 to 192 h post‐infusion. Total radiance efficiency decreased rapidly within the first 48 h (Figure S1a) to near background levels. At 192 h post‐infusion (more than a week), no evidence of DiR‐labeled MSCs signals were detected in any of the organs and tissues analyzed (data not shown). Most of the total radiance efficiency was in the liver at 2 h post‐infusion, slightly higher in DSS‐induced colitic mice than in healthy mice, with no significant differences between WT‐MSCs and CXCR4‐IL10‐MSCs (Figure S1a–c,e). A reduced total radiance efficiency was detected in other organs and tissues (ranging from 1% to 3%), with no signal detection in the caudal and mesenteric lymph nodes, heart, reproductive organs, or thymus (Figure S1e).

A slightly lower total radiance efficiency was found in the bone marrow of DSS‐induced colitic mice injected with CXCR4‐IL10‐MSCs compared to those injected with WT‐MSCs (6.8[4.9–9.4] vs. 12.3[5.1–17.8] × 10^9^ [p/s]/[μW/cm^2^]), respectively (Figure S2c). No differences were observed in healthy mice (Figure S2b).

**FIGURE 2:**
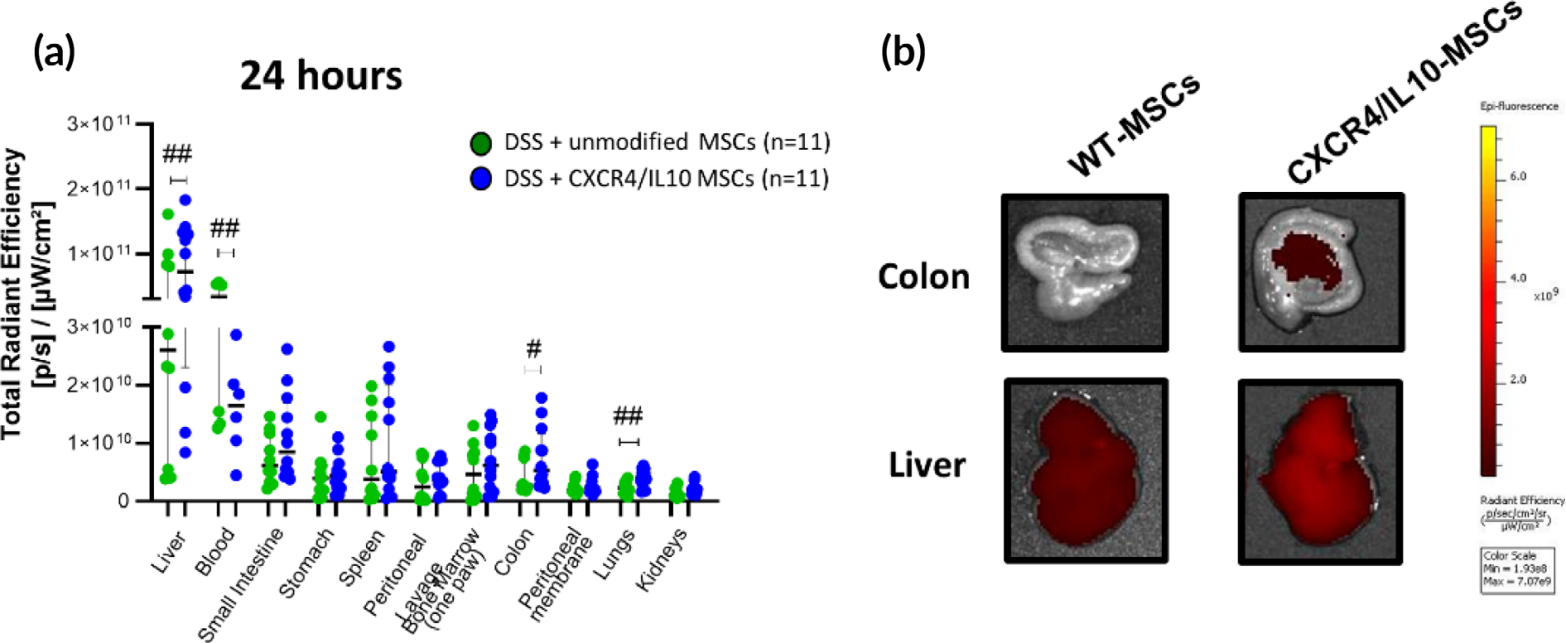
Biodistribution of WT‐ and CXCR4‐IL10‐MSCs at 24 h post‐infusion in DSS‐induced colitic mice. (a) Total radiant efficiency ([p/s]/[μW/cm^2^]) represented by median and interquartile range (p75, upper edge; p25, lower edge; p50, midline) in liver, blood, small intestine, stomach, spleen, peritoneal cavity lavage, bone marrow (one paw) lavage, colon, peritoneal serous membrane, lungs and kidneys. (b) Representative images of total radiant efficiency in the colon (top) and liver (bottom) at 24 h after intraperitoneal administration of WT‐ (green, left, *n* = 11) and CXCR4‐IL10 (blue, right, *n* = 11) Dir‐labeled MSCs on day 5 of the 7‐day DSS cycle in colitic mice. Significance was analyzed by the Mann–Whitney *U* test and represented by ^#^ *p* < 0.05 and ^##^ *p* < 0.01 DSS + CXCR4‐IL10‐MSCs versus DSS + WT‐MSCs. Results correspond to seven independent experiments.

A significantly higher total radiance efficiency was found in the liver (7.1[2.3–13.2] vs. 2.6[0.5–8.7] × 10^10^ [p/s]/[μW/cm^2^]), colon (5.2[3.0–11.6] vs. 2.8[2.1–7.6] × 10^9^ [p/s]/[μW/cm^2^]) and lungs (4.0[2.0–5.4] vs. 2.3[1.4–2.9] × 10^9^ [p/s]/[μW/cm^2^]) in DSS‐induced colitic mice injected with CXCR4‐IL10‐MSCs compared to those injected with WT‐MSCs at 24 h post‐infusion (Figure 2a). Conversely, lower total radiance efficiency was found in the peripheral blood in DSS‐induced colitic mice injected with CXCR4‐IL10‐MSCs compared to those injected with WT‐MSCs (1.6[0.9–2.2] vs. 3.3[1.3–5.3] × 10^10^ [p/s]/[μW/cm^2^], Figures 2a,b and S1). After 48 h, no significant differences in total radiance efficiency were observed between DSS‐induced colitic mice injected with WT‐MSCs and those injected with CXCR4‐IL10‐MSCs across the organs analyzed (Figure S1d), suggesting that the genetically‐induced expression of CXCR4 in Ad‐MSCs accelerates the trafficking of the CXCR4‐IL10‐MSCs to the target tissues although the biodistribution pattern in the tissues was similar to WT‐MSCs.

### 2.3 CXCR4‐IL10‐MSCs enhance the regulatory/inflammatory balance in the colon of colitic mice compared with WT‐MSCs

To investigate the mechanisms contributing to the improved efficacy of CXCR4‐IL10‐MSCs over WT‐MSCs, we monitored the progression of inflammation by means of the hematological analysis of peripheral blood samples. As expected, colitic mice exhibited a significant increase in white blood cells (granulocytes, monocytes, and lymphocytes, Figure S2a‐d), along with elevated platelet counts and decreased red blood cells and hemoglobin levels (Figure S2e‐g). On day 6, CXCR4‐IL10‐MSCs treated colitic mice showed an increase in monocyte percentage compared to WT MSC‐treated and untreated colitic mice, as well as to healthy controls (Figure S2c), which is consistent with our previous findings in a model of collagen‐induced arthritis.^32^

**FIGURE 3:**
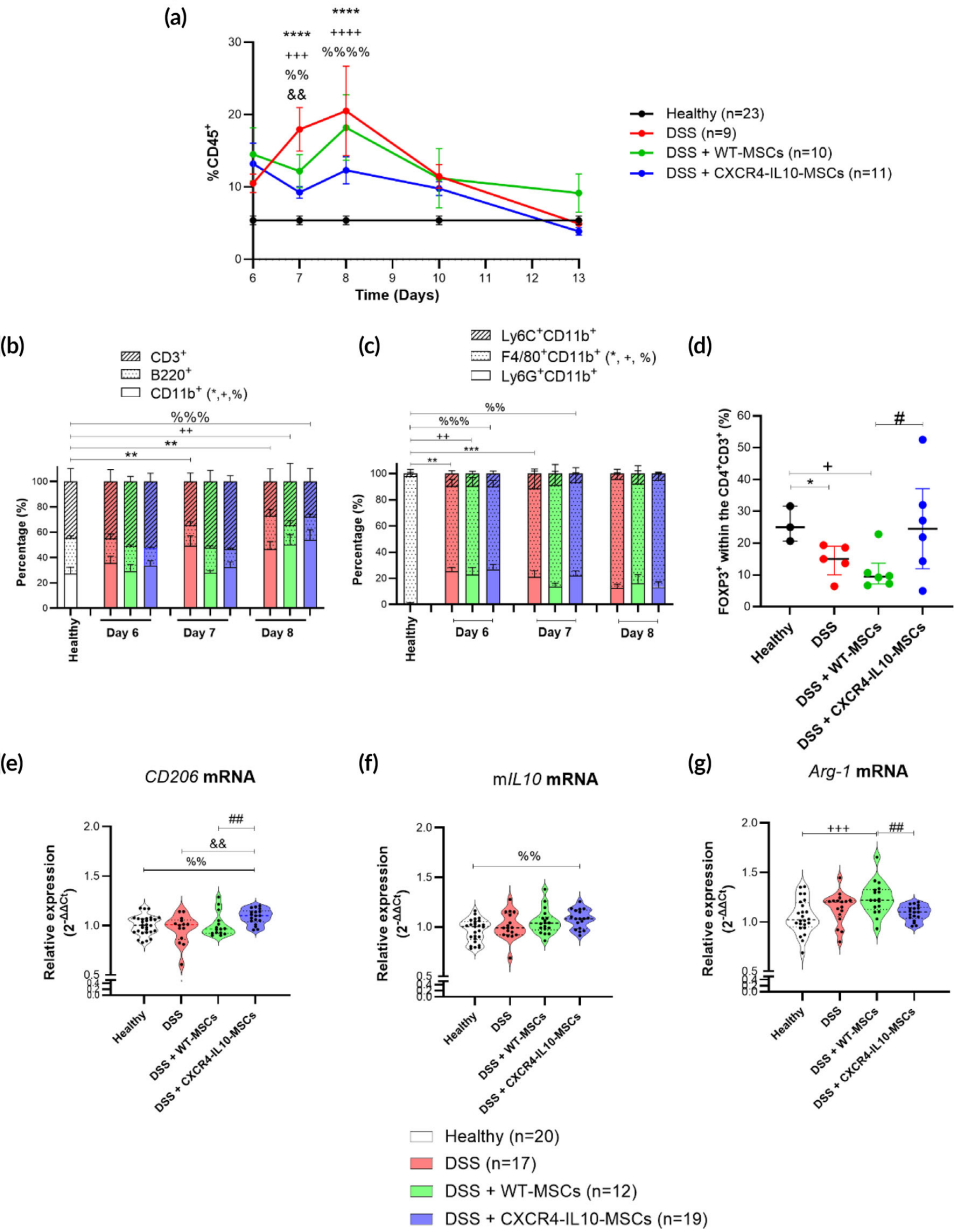
Immune response analysis in the colon during the first 7‐day DSS cycle with WT‐ and CXCR4‐IL10‐MSCs treatment. (a) Kinetics of CD45+ leukocyte infiltration, (b) types of leukocytes identified by CD3, B220 and CD11b expression and (c) myeloid cell populations (within CD45+ cells identified by Ly6C, F4/80 and Ly6G) measured by flow cytometry from day 6 to day 13 of 7‐day DSS cycle. (d) Regulatory T cells (within CD4 + CD3+, identified by FOXP3) on day 13 of 7‐day DSS cycle in the colon expressed as mean and standard error of the mean. Healthy (*n* = 23), WT‐ (*n* = 10) and CXCR4‐IL10‐MSC‐treated (*n* = 11) and untreated (*n* = 9) colitic mice. Violin plots show the median and interquartile range of mRNA levels of CD206 (e), mouse IL10 (f) and arginase (Arg) 1 (g) measured by qRT‐PCR on day 8 of the 7‐day DSS cycle in colon. Healthy (*n* = 23), WT‐ (*n* = 18) and CXCR4‐IL10‐MSC‐treated (*n* = 18) and untreated (*n* = 17) colitic mice. Significance was analyzed using the Mann–Whitney *U* test, represented by *p < 0.05, **p < 0.01, ***p < 0.001 and ****p < 0.0001 DSS versus heathy; ^+^ *p* < 0.05, ^++^ *p* < 0.01, ^+++^ *p* < 0.001 and ^++++^ *p* < 0.0001 DSS + WT‐MSCs versus healthy; ^%^ *p* < 0.05, ^%%^ *p* < 0.01, ^%%%^ *p* < 0.001 and ^%%%%^ *p* < 0.0001 DSS + CXCR4‐IL10‐MSCs versus healthy; ^&&^ *p* < 0.01 DSS + CXCR4‐IL10‐MSCs versus DSS and ^#^ *p* < 0.05 and ^##^ *p* < 0.01 DSS + CXCR4‐IL10‐MSCs versus DSS + WT‐MSCs. Results correspond to seven independent experiments.

Flow cytometry analysis performed revealed that the treatment with CXCR4‐IL10‐MSCs reduced CD45^+^ leukocyte infiltration significantly (9.2 ± 0.0% on day 7 and 12.3 ± 0.0% on day 8) in comparison to untreated DSS‐induced colitic mice (18.0 ± 0.0% on day 7 and 20.5 ± 0.0% on day 8), while WT‐MSC treatment resulted in a modest decrease in CD45^+^ leukocyte infiltration (12.2 ± 0.0% on day 7, Figure 3a). No significant differences in the inflammatory/regulatory cytokine balance were observed following treatment with either CXCR4‐IL10‐MSCs or WT‐MSCs (Figure S2h‐n).

A significant increase of myeloid cells within CD45^+^ cells, defined by CD11b expression, was observed in DSS‐induced colitic mice compared to healthy controls (Figure 3b). MSC infusion in these colitic mice, either WT or CXCR4‐IL10‐MSCs, restored CD11b levels (Figures 3c and S3). Although no differences in myeloid populations (monocytes, neutrophils, and macrophages) were seen following treatment with either MSC types, CXCR4‐IL10‐MSC‐treated colitic mice showed a significant increase in CD206 expression (Figure 3e) and mouse IL10 mRNA levels (Figure 3f) compared to healthy, untreated, and WT MSC‐treated colitic mice. This suggests that increased frequencies of regulatory M2 macrophages colonized the colon. Of interest, DSS‐colitic mice treated with WT‐MSCs showed a significant increase in the expression of Arg‐1 levels compared with colitic CXCR4‐IL10‐MSCs‐treated mice and healthy mice (Figure 3g).

No differences in the percentage of T (CD4^+^CD3^+^ and CD8^+^CD3^+^) or B cell populations (B220^+^ cells) were observed in untreated or MSC‐treated colitic mice compared to healthy controls (Figures 3b and S2). During the recovery phase, on day 13, a significant increase in regulatory T cells was observed cells (FOXP3 marker) within CD4^+^ T cell population was observed in CXCR4‐IL10‐MSC‐treated colitic mice compared to healthy mice and to WT‐MSC‐treated colitic mice (Figures 3d and S4).

No macroscopic changes in thymuses or lymph nodes were observed, suggesting minimal impact on immune competence under these conditions (Figure S1a).

In conclusion, these data show that compared to WT‐MSCs, CXCR4‐IL10‐MSCs enhance the regulatory/inflammatory balance in the colons of DSS‐induced colitic mice.

### 2.4 Enhanced long‐term protective effects of CXCR4‐IL10‐MSC‐based therapy in colitic mice

Building on our previous findings^31^ and recognizing the need for therapies that promote long‐term protection in IBD, a second DSS cycle was repeated in colitic mice previously treated or not with the different types of MSCs. We repeated the second DSS challenge after a latency period of 12 weeks when all groups of mice reached normal levels of PB parameters (white blood cells, granulocytes, monocytes, lymphocytes, platelets, red blood cells, and hemoglobin; Figure S5a‐g) and also when body weights (Figure S5h), colon histology (Figure S5i), and leukocyte levels (Figure S5j,k) were normalized, which mimicked remission phases seen in IBD patients^33^, ^34^, ^35^ and supported the therapy's favorable safety profile in the long term.

**FIGURE 4:**
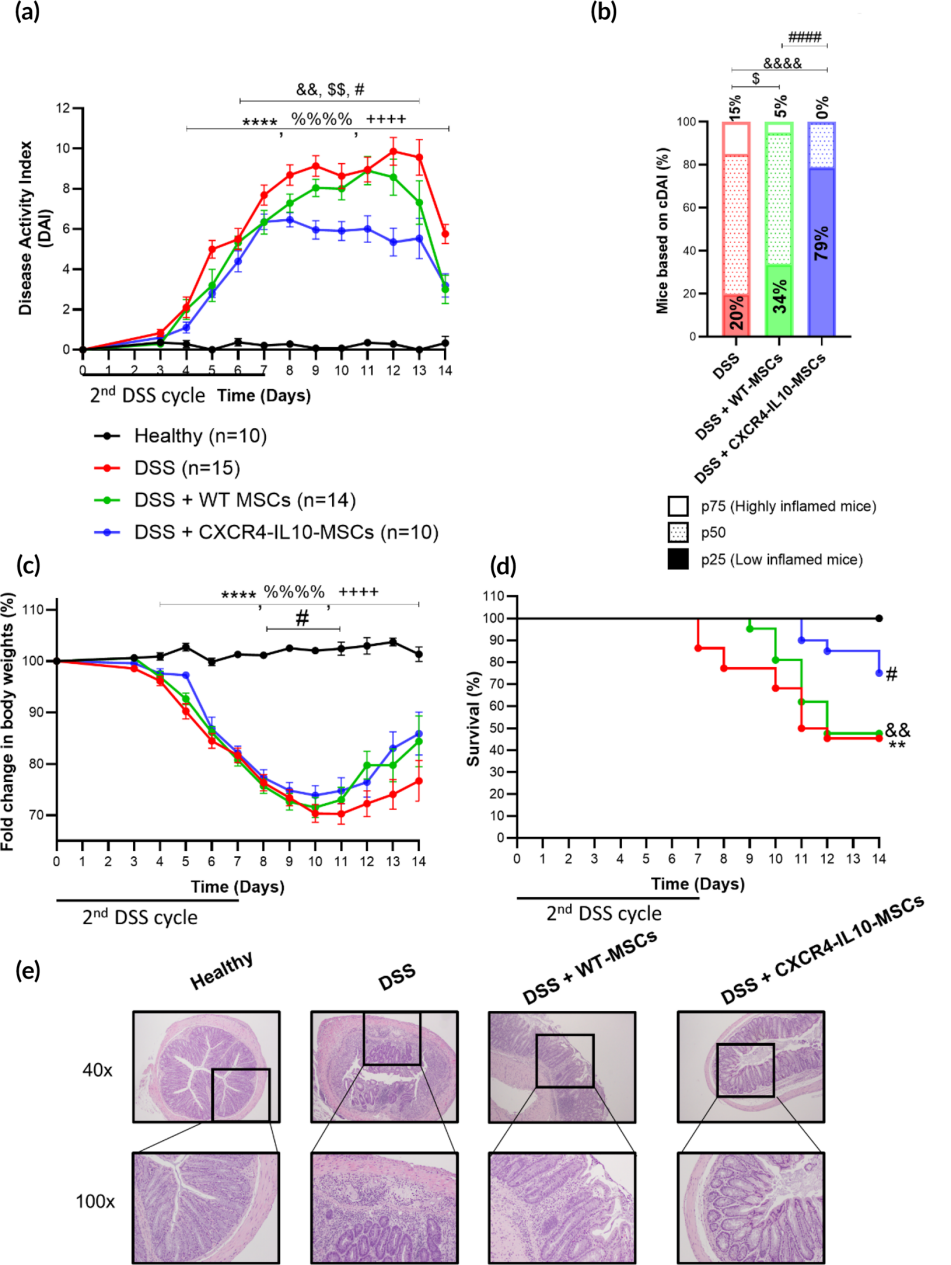
Colitis status of mice during a second 7‐day DSS cycle following a 12‐week latency period. (a) Disease activity index (DAI), (b) contingency graph showing percentages of colitic mice (WT‐ and CXCR4‐IL10‐MSC‐treated or untreated) classified by DAI percentiles, (c) fold change in body weights and (d) survival during the second 7 day‐DSS cycle. (e) Representative images of colon tissue (magnification 40× and 100×) on day 8 after DSS treatment. Data are presented as mean ± standard error of the mean for DAI and fold change in body weights. Survival data are shown as percentages. Significance was analyzed by the Mann–Whitney *U* test for DAI and fold change in body weights, log rank test for survival and Fisher's test for the contingency graph. Significant levels are represented by **p < 0.01 and ****p < 0.0001 DSS versus heathy, $*p* < 0.05 and $$*p* < 0.01 DSS + WT‐MSCs versus DSS, ^&&^ *p* < 0.01 and ^&&&&^ *p* < 0.0001 DSS + CXCR4‐IL10‐MSCs versus DSS and ^#^ *p* < 0.05 and ^####^ *p* < 0.0001 DSS + WT‐MSCs versus + CXCR4‐IL10‐MSCs. Healthy, *n* = 10; DSS, *n* = 15, DSS + WT‐MSCs, *n* = 14 and DSS + CXCR4‐IL10‐MSCs, *n* = 10. Results correspond to three independent experiments.

We observed that the re‐challenge of a second DSS cycle in mice that had been treated with CXCR4‐IL10‐MSCs during the first DSS cycle exhibited reduced signs of colitis as shown by significantly improved DAI scores and body weights (Figure 4a,c), as well as the overall survival rates (Figure 4d) compared to mice that were treated with WT‐MSCs during the first DSS cycle. Moreover, a significant increase in the percentage of responder mice (79%) was observed in the CXCR4‐IL10‐MSC‐treated group compared to the WT‐MSC‐treated group (34%) and the untreated colitic mouse group (20%, Figure 4b). Additionally, histological analyses showed a better‐preserved colon morphology and reduced leukocyte infiltration in CXCR4‐IL10‐MSC‐treated colitic mice compared to WT‐MSC‐treated and untreated colitic groups of mice at day 9 (Figure 4e).

These results confirm that MSCs engineered to stably express CXCR4 and IL10 represent a promising MSC‐based cell therapy with improved long‐term protection upon recurrent inflammatory challenges with respect to WT‐MSCs.

**FIGURE 5:**
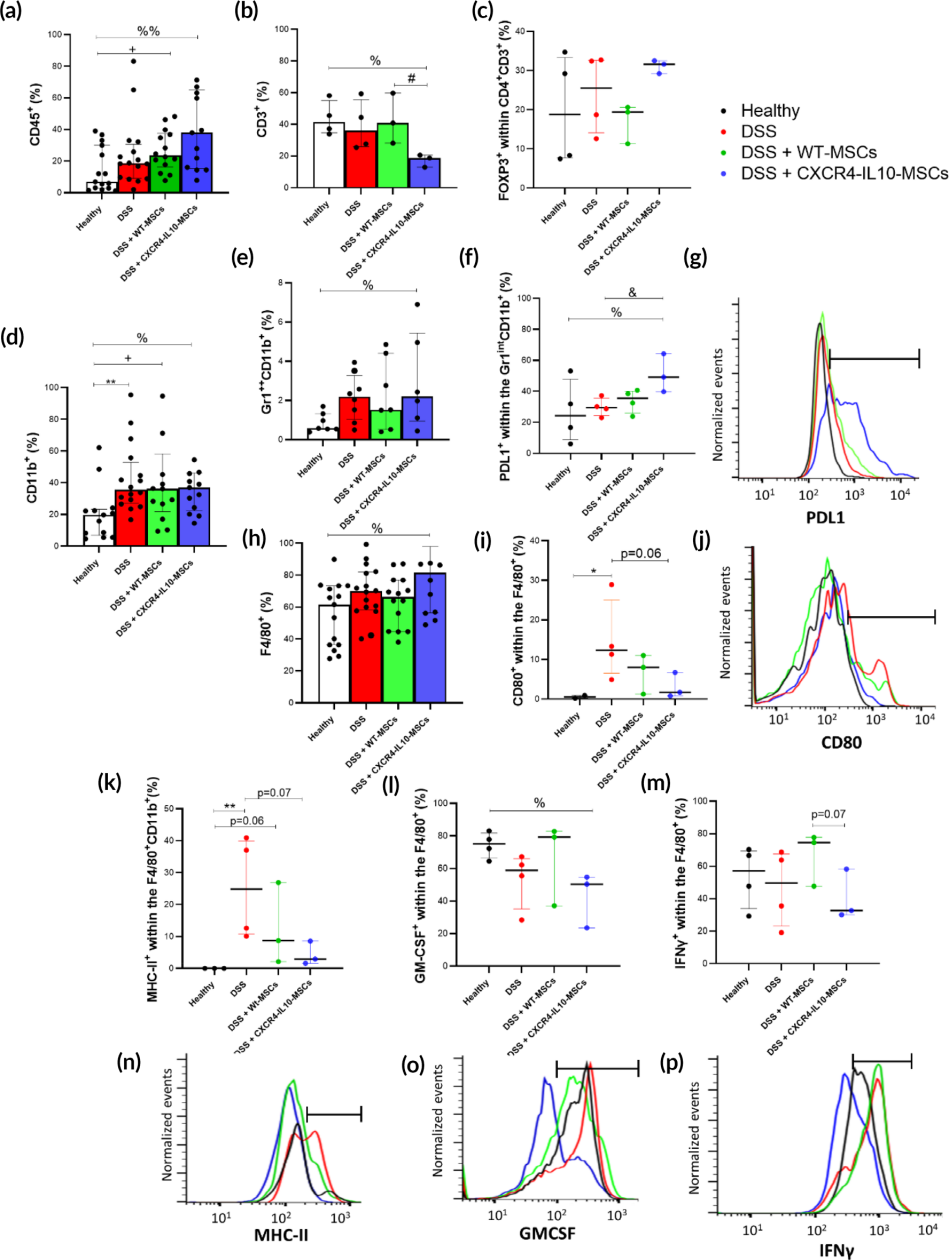
Immune responses in the colon of healthy, untreated, WT‐ and CXCR4‐IL10‐MSC‐treated colitic mice on day 7 of a 2nd 7‐day DSS cycle following a 12‐week latency. Percentages of CD45+ (a), CD3+ (b), FOXP3+ (within CD4 + CD3+, c), CD11b + (d), Gr1brightCD11b + (e) and F4/80+ (h) cells. Expression and representative histograms of PDL‐1 within Gr1intCD11b + (f and g) and CD80 (i and j), MHC‐II (k and n), GM‐CSF (l and o) and IFNÎ^3^ (m and p) within F4/80+ in the colon of healthy (black line, *n* = 3–15), untreated (red line, *n* = 4–16) and WT‐ (green line, *n* = 4–16) and CXCR4‐IL10‐ (blue line, *n* = 3–16) MSC‐treated colitic mice. Black bar refers to positive expression. Data are presented as medians and interquartile ranges (p75, upper edge; p25, lower edge; p50, midline) of the percentages. Significance was analyzed using the Mann–Whitney *U* test, represented as number if *p* = 0.1–0.05, *p < 0.05 and **p < 0.01 DSS versus heathy; +*p* < 0.05 DSS + WT‐MSCs versus healthy; ^%^ *p* < 0.05 and ^%%^ *p* < 0.01 DSS + CXCR4‐IL10‐MSCs versus healthy; ^&^ *p* < 0.05 DSS + CXCR4‐IL10‐MSCs versus DSS and ^#^ *p* < 0.05 DSS + CXCR4‐IL10‐MSCs versus DSS + WT‐MSCs. Results correspond to two independent experiments.

We investigated changes in colon cell parameters in these mice to try to understand the mechanisms behind the improved long‐term protective effects observed with engineered MSCs in the DSS‐induced colitis model. A significant increase in CD45^+^ leukocytes was first observed in the colon of DSS re‐challenged colitic mice treated either with CXCR4‐IL10‐MSCs or WT‐MSCs compared to untreated colitic mice (Figure 5a). Within the CD45^+^ population, a significant decrease in CD3^+^ cells was observed in CXCR4‐IL10‐MSC‐treated compared to WT‐MSC‐treated colitic mice as well as healthy mice (Figure 5b). Among the CD4^+^CD3^+^ cell population, a tendency to increase the frequencies of regulatory T cells (identified by FOXP3 expression) was observed in CXCR4‐IL10‐MSC‐treated colitic mice compared to healthy, untreated, and WT‐MSC‐treated colitic mice (Figure 5c). No differences in B cells (identified by the B220 marker) were observed among the different groups of mice (Figure S6a).

In contrast to T and B cell populations, a significant increase in myeloid cells, measured by CD11b expression, was observed in both untreated and MSC‐treated colitic mice (CXCR4‐IL10‐MSCs or WT‐MSCs) compared to healthy controls (Figure 5d). Regarding CD11b^+^ myeloid cells, increased populations expressing Gr1^bright^ (Figure 5e) and F4/80 (Figure 5h) were found in the colon of CXCR4‐IL10‐MSC‐treated colitic mice compared to healthy, untreated, and WT‐MSC‐treated colitic mice, without significant differences in Gr1 intermediate‐expressing cells (Figure S6b). Interestingly, within the Gr1^bright^CD11b^+^ (Figure 5f,g) and F4/80^+^ populations (Figure S6c,d), a higher expression of programmed cell death ligand (PDL) 1 was found in CXCR4‐IL10‐MSC‐treated colitic mice compared to healthy and WT‐MSC‐treated and untreated colitic mice. In contrast, within those F4/80^+^ cells, lower expression levels of inflammatory profile markers such as CD80 (Figure 5i,j), MHC‐II (Figure 5k,n), GM‐CSF (Figure 5l,o), and IFNγ (Figure 5m,p) were found in CXCR4‐IL10‐MSC‐treated colitic mice compared to untreated, healthy, and WT‐MSC‐treated colitic mice. Additionally, IFNγ mRNA levels were decreased in both groups of MSC‐treated colitic mice compared to untreated colitic mice (Figure S6e). No clear differences were observed in the other inflammatory cytokine mRNA levels (TNFα, mIL6, COX 2, and iNOS, Figure S6f‐i).

In summary, CXCR4‐IL10‐MSC treatment during the first DSS cycle enhances the regulatory/inflammatory ratio in the colon. The long‐term protection observed in these mice is accompanied by reducing the frequency of inflammatory F4/80^+^CD11b^+^ cells and increasing PDL1‐expressing Gr1^int^CD11b^+^ myeloid cells and regulatory FOXP3‐expressing T cells.

**FIGURE 6:**
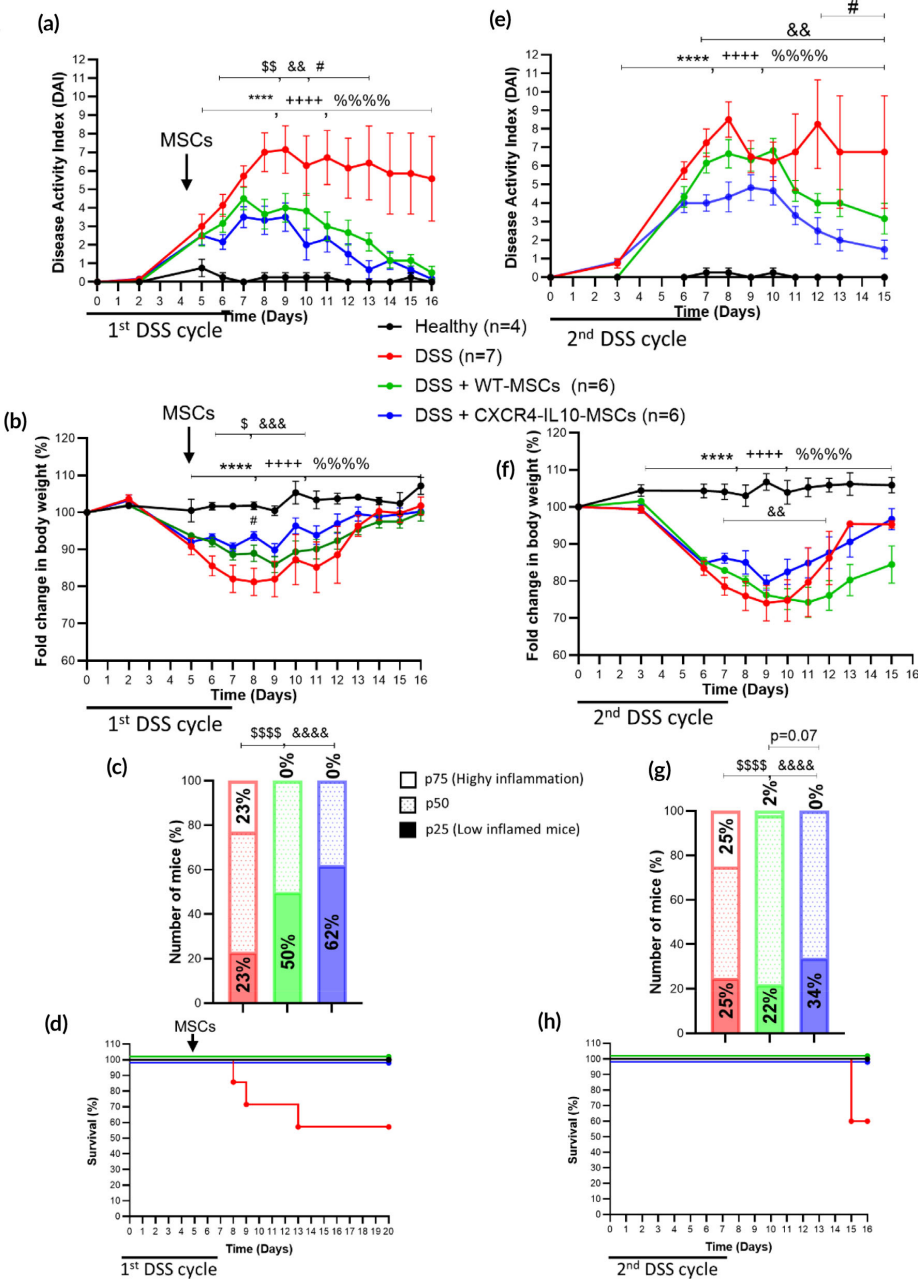
Colitis status of Rag‐deficient mice during the first and second 7‐day DSS cycles. Disease activity index (DAI) during the 1st (a) and 2nd (e) 7‐day DSS cycles, fold change in body weights during the 1st (b) and during 2nd (f) 7‐day DSS cycles, contingency graph showing the percentage of colitic mice classified by cumulative DAI percentiles during the 1st (c) and 2nd (g) 7‐day DSS cycles and survival during the 1st (d) and 2nd (h) 7‐day DSS cycles. Data are presented as mean ± standard error of the mean for DAI and fold change in body weights. Survival data are presented as percentages. Significance was analyzed using the Mann–Whitney *U* test for DAI and fold change in body weights, log rank test for survival and Fisher's test for the contingency graph. Significant levels are represented by ****p < 0.0001 DSS versus heathy; ^++++^ *p* < 0.0001 DSS + WT‐MSCs versus DSS; ^%%%%^ *p* < 0.0001 DSS + CXCR4‐IL10‐MSCs versus DSS; ^$^ *p* < 0.05, ^$$^ *p* < 0.01 and ^$$$$^ *p* < 0.0001 DSS + WT‐MSCs versus DSS; ^&&^ *p* < 0.01, ^&&&^ *p* < 0.001 and ^&&&&^ *p* < 0.0001 DSS + CXCR4‐IL10‐MSCs versus DSS and ^#^ *p* < 0.05 DSS + WT‐MSCs versus + CXCR4‐IL10‐MSCs. Healthy, *n* = 4; DSS, *n* = 7, DSS + WT‐MSCs, *n* = 6 and DSS + CXCR4‐IL10‐MSCs, *n* = 6. Representative experiment of three independent experiments.

Previous studies by our group have identified that the long‐term anti‐inflammatory effects mediated by WT‐MSCs in experimental colitis were induced by an anti‐inflammatory innate immune memory independent of the adaptive immune responses.^31^ Therefore, to investigate the role of the innate immune system in the improved therapeutic efficacy of CXCR4‐IL10‐MSCs over WT‐MSCs, we developed a DSS‐induced colitic model using Rag‐2 deficient mice, which lack T and B cell responses. As shown in Figure 6, CXCR4‐IL10‐MSCs showed significantly improved therapeutic effects compared to WT‐MSCs, as shown by a decrease in the DAI (Figure 6a) and a reduced loss in body weight following DSS treatment (Figure 6b). Furthermore, based on the cumulative DAI (Figure 6c), a significant increase in the proportion of responder colitic Rag‐2 deficient mice (62%) was observed in CXCR4‐IL10‐MSC‐treated compared to WT‐MSC‐treated colitic Rag‐2 deficient mice (50%).

After a latency period of 12 weeks and a second 7‐day DSS challenge, CXCR4‐IL10‐MSCs also showed an improved long‐term protective effect compared to WT‐MSCs, as indicated by a reduction in the DAI (Figure 6e). The cumulative DAI (Figure 6g) also revealed a higher percentage of responder mice (34%) in the CXCR4‐IL10‐MSC‐treated colitic mice compared to WT‐MSC‐treated colitic mice (22%). The overall survival of the DSS‐colitic Rag‐2 deficient mice was well preserved in the WT‐MSCs and CXCR4‐IL10‐MSC‐treated colitic Rag‐2 deficient mice with respect to untreated DSS‐colitic mice both during the first DSS inflammatory challenge and (55% survival in untreated colitic Rag‐2 deficient mice vs. 100% survival in WT‐MSCs and CXCR4‐IL10‐MSC‐treated colitic Rag‐2 deficient mice; Figure 6d). Similarly, upon the recurrent DSS challenge 12 weeks after the infusion of the WT and CXCR4‐IL10‐MSCs, a 60% survival in untreated colitic Rag‐2 deficient mice versus 100% survival in WT‐MSCs and CXCR4‐IL10‐MSC‐treated colitic Rag‐2 deficient mice was observed (Figure 6h).

These results pointed out that the improved therapeutic effects of CXCR4‐IL10‐MSCs are at least in part mediated by an induction of an anti‐inflammatory innate immune memory response that also accounts for the long‐term protective effects of CXCR4‐IL10‐MSCs in a mouse model of experimental colitis.

## 3 DISCUSSION

Mesenchymal stromal cell‐based therapy has emerged as a promising approach for treating inflammatory diseases thanks to their immunomodulatory and tissue regenerative capabilities. Despite these advantages, the moderate efficacy observed in most phase III clinical trials has limited their widespread clinical application. Our previous work demonstrated the enhanced therapeutic potential of CXCR4‐IL10‐engineered MSCs in a mouse model of pad inflammation^11^ and also in a humanized mouse model of GvHD.^10^ These findings encouraged us to investigate whether these engineered MSCs could provide an improved therapeutic strategy for patients with inflammatory bowel disease (IBD).

To investigate this, we employed the widely used dextran sulfate sodium (DSS)‐induced colitis mouse model, which has been instrumental in advancing our understanding of IBD pathogenesis and treatment.^36^, ^37^ Genetically engineering MSCs enhances their migration towards inflamed tissues thanks to the constitutive CXCR4 expression, which coupled with the immunomodulatory effects of IL10, this approach represents a viable next‐generation MSC strategy.^38^

Based on our prior studies showing that MSC‐based therapies can effectively modulate intestinal inflammation when administered early in the disease course, we infused WT and CXCR4‐IL10‐MSCs during the acute inflammatory phase of DSS‐induced colitis. The infusion of engineered MSCs significantly reduced and delayed colon inflammation, increasing the proportion of responder mice compared to those treated with unmodified MSCs. We used human clinical score parameters for assessing intestinal inflammation, thus highlighting the translational potential of these findings into clinical practice.^39^

In DSS‐induced colitis, colon inflammation is characterized by severe colon leukocyte infiltration, primarily involving monocytes and granulocytes.^40^, ^41^ Our results indicated that engineered MSCs markedly reduced leukocyte infiltration in the colon of DSS‐induced colitic mice. Additionally, we observed elevated levels of M2 macrophage markers in the colon lamina propria, likely driven by increased local IL10 production from the higher number of engineered MSCs reaching the inflamed tissue compared to unmodified MSCs.^42^, ^43^ The known effects of IL10 on monocyte and macrophage subsets^44^ promoting M2 polarization^45^ further explained the observed reduction in leukocyte infiltration and the shift towards a regulatory profile. Ultimately, the presence of M2 macrophages, together with high TGFβ levels produced by local MSCs,^10^ likely contributes to the favorable regulatory/inflammatory balance and the induction of regulatory T cells observed by day 13.

Although similar biodistribution patterns were observed for both CXCR4‐IL10‐MSCs and WT‐MSCs, which is consistent with the dependence of biodistribution on the route of administration,^46^, ^47^, ^48^, ^49^, ^50^, ^51^, ^52^, ^53^ at 24 h upon infusion of CXCR4‐IL10‐MSCs, increased trafficking to tissues and organs was detected compared to WT‐MSCs. This enhanced trafficking may be attributed to elevated SDF‐1 levels in inflamed tissues,^54^, ^55^ including in the colons of IBD patients,^17^ confirming that the genetically induced expression of CXCR4 represents an improved strategy to enhance MSC‐based therapy efficacy in IBD.

IL10 therapy has previously been shown to alleviate IBD‐like symptoms in both preclinical^19^ and clinical settings.^20^, ^21^ However, the repeated systemic use of high doses of IL10 in clinical trials led to significant adverse effects^21^, ^22^, ^23^ such as anemia, thrombocytopenia, flu‐like symptoms, fever, and hypotension. These toxicities were attributed, at least in part, to IL10's pleiotropic effects on the immune and hematopoietic system. In contrast, in our studies, hematological parameters were normalized at day 60 post‐infusion, and no tumors were observed in any treated animal. Furthermore, no macroscopic changes were observed in the thymus or peripheral lymph nodes in any case, supporting the favorable safety profile with respect to oncogenicity and systemic immunosuppression. Together, these findings suggest a novel strategy to boost local IL10 levels without inducing any systemic side effects. Consistent with this, we observed a rapid decline of DiR‐CXCR4‐IL10‐MSC signals within the first 48 h post‐infusion, which almost disappeared at 192 h. This transient IL10 expression may not only reduce the risk of thrombocytopenia or anemia but also minimize systemic immunosuppression and its associated risks, including opportunistic infections or tumor‐promoting effects. Our data therefore indicate that the enhanced therapeutic effects of CXCR4‐IL10‐MSCs, relative to WT‐MSCs, are most likely mediated by early paracrine signaling rather than sustained MSC engraftment. Finally, hematological analyses revealed a transient increase in the proportion of circulating monocytes in CXCR4‐IL10‐MSC‐treated colitic mice compared to unmodified MSC‐treated animals. This observation aligns with previous studies^32^ showing that MSCs promote the induction of myeloid cells with regulatory phenotypes, which are necessary for the therapeutic benefits observed.^56^, ^57^, ^58^, ^59^, ^60^

Although we observed a rapid decline in DiR‐labeled MSC signals within the first 48 h post‐infusion, consistent with findings from our own group^48^ and from other groups,^61^ the recurrent inflammatory challenge with DSS revealed a significantly greater attenuation of intestinal inflammation in mice previously treated with CXCR4‐IL10‐MSCs as compared with those infused with WT‐MSCs. This was evidenced by improved survival rates and a higher proportion of responder mice. These results highlight the long‐term protective effects of engineered MSCs relative to WT‐MSCs, which is particularly relevant given the recurrent inflammatory nature of IBD.^62^

Long‐term analyses of the colon revealed increased expression of the PDL1 marker within Gr1^int^CD11b^+^ cells in mice treated with CXCR4‐IL10‐MSCs, suggesting the induction of immunosuppressive myeloid cells, which are critical for immune regulation. Gr1^+^CD11b^+^ cells comprise both classical (CMs) and non‐classical monocytes (NCMs), each contributing to the generation of tissue‐resident macrophage populations.^63^, ^64^ Mice treated with CXCR4‐IL10‐MSCs exhibited reduced expression of CD80, GM‐CSF, and IFNγ together with increased PDL1 expression in F4/80^+^CD11b^+^ myeloid cells, indicating a phenotypic shift from pro‐inflammatory M1 macrophages towards regulatory M2 macrophages. This polarization is consistent with the establishment of an anti‐inflammatory microenvironment that supports tissue repair and immune tolerance. Together, these effects likely contribute to the enhanced tolerance and long‐term protection observed against recurrent DSS‐induced colitis, highlighting the importance of early paracrine signaling by engineered MSCs in orchestrating sustained immune regulation. CMs and NCMs arise from granulocyte‐monocyte progenitors (GMP‐Mo) and monocyte‐dendritic cell precursors (MDP‐Mo). NCMs, which have longer lifespans than CMs, are particularly important because they originate from MDPs and express higher levels of PDL1, thereby enhancing their immunosuppressive activity on effector T cells.^65^ The localized increase in IL10 likely promotes an anti‐inflammatory environment^66^ by inducing a durable epigenetic fingerprint in monocytes favoring the generation of circulating monocytes that contribute to enhanced tolerance against recurrent DSS‐induced colitis. Additionally, the CXCR4‐mediated homing of MSCs to inflamed sites may amplify IL10's local effects at the inflamed colon, enabling MSC interactions with lamina propria monocytes and macrophages, which secrete other immunomodulatory factors and drive M2 polarization.^64^ The increased number of monocytes observed in the peripheral blood of CXCR4‐IL10‐MSC‐treated mice may therefore reflect both IL10‐driven imprinting and MSC‐mediated conditioning. To dissect the relative contribution of these mechanisms, future experiments such as specific human IL10 blockade or the use of MSCs engineered solely with CXCR4 will help to determine whether long‐term protection is mediated primarily by IL10 signaling, improved MSC homing, or a combination of both. These results highlight the anti‐inflammatory context established in mice treated with engineered MSCs, which is characterized by a significant decrease in IFNγ and an increase in IL6 and COX‐2. In this environment, IL6 serves as a modulator that, via COX‐2, promotes PGE2 production, a key mediator of tissue regeneration, further favoring M2 macrophage polarization.^67^

Building on our previous studies^31^ and to further elucidate the role of the immune system in MSC‐mediated effects in colitic mice, we conducted experiments using Rag‐2^−/−^ mice, which lack T and B cell responses.^31^ DSS‐induced colitic Rag‐2^−/−^ mice treated with CXCR4‐IL10‐MSCs exhibited significantly reduced body weight loss, improved survival, and lower disease activity indices both in the short‐ and long‐term compared to Rag‐2^−/−^ mice treated with unmodified MSCs. These findings suggest that T and B cells are not primary mediators of the enhanced therapeutic benefits conferred by CXCR4‐IL10‐MSCs and highlight the pivotal role of innate immune memory induced by genetically engineered MSC therapy.

While alternative strategies have been developed to enhance the immunomodulatory and regenerative properties of MSCs, our study is the first to utilize dual‐engineered CXCR4‐IL10‐MSCs to simultaneously enhance MSC trafficking to inflamed tissues and boost their immunomodulatory functions in preclinical colitis models. Fu et al. demonstrated improved efficacy with intravenously infused xenogeneic CX3CR1‐IL25‐MSCs in colitis models; however, their study did not evaluate long‐term outcomes. They highlighted the role of IL25 to enhance the anti‐inflammatory effects of MSCs and CX3CR1 to promote MSC migration to inflamed tissues.^68^ It has been demonstrated that IBD patients express high levels of CX3CL1 in the colon^69^ and that the exogenous administration of a high dose of IL25 protects against colitis in a similar colitis model.^70^ Additionally, Nan et al. engineered syngeneic MSCs to express CXCR4 and IL35 in a TNBS‐induced colitis rat model, reporting improved efficacy. However, the long‐term effects of the MSC‐based therapy were not assessed.^71^ Importantly, unlike IL10, neither IL25 nor IL35 has been clinically employed as an anti‐inflammatory treatment to date, highlighting the translational advantage of IL10‐based therapies.

Overall, the marked enhancement of both short‐ and long‐term efficacy observed with CXCR4‐IL10‐MSCs following a colon‐inflammation challenge strongly supports the therapeutic potential of these cells for patients with IBD.

## 4 MATERIALS AND METHODS

### 4.1 Mice

C57BL/6JRj and C57BL/6N‐Rag2Tm1/CipheRj mice were obtained from Janvier.

### 4.2 Generation, expansion and characterization of genetically‐modified MSCs expressing CXCR4 and IL10

Adipose‐derived mesenchymal stromal cells (Ad‐MSCs) were isolated from human adipose tissue and transduced with codon‐optimized sequences of CXCR4 and IL10 genes. Codon‐optimized sequences were cloned into a bicistronic lentiviral vector under the human PGK promoter, as described in^10^ (Figure S7). Human adipose tissue samples were obtained following informed consent approved by the Spanish Ethics Committee (IIS‐Fundación Jiménez Díaz, Madrid, Spain). In vitro characterization demonstrated that the modification of the MSCs with the bicistronic lentiviral vector did not alter their immunophenotype or their ability to differentiate into bone and adipose tissues when compared to unmodified mesenchymal stromal cells (WT‐MSCs), according to the criteria established by the International Society of Cellular Therapy (ISCT) for mesenchymal stromal cells.^10^ For both in vitro and in vivo studies, MSCs were used at passages 4–8.

### 4.3 Colitis induction and experimental design

Different concentrations of dextran sulfate sodium (DSS; 36,000–50,000 MW, MP Biomedicals, Irvine CA USA) were administered in drinking water ranging from 2.5% to 3% for 7 days ad libitum.^40^ Based on our previous findings,^31^ a single dose of either WT‐ or CXCR4‐IL10‐MSCs (3 × 10^6^ cells/mouse) was intraperitoneally (IP) infused on day 5. For long‐term evaluation, a re‐challenge was conducted with a 7‐day cycle of DSS in drinking water after a latency period of 12 weeks.

The colitis score or disease activity index (DAI) was assessed using the following criteria: (1) Body weight loss (0: no loss; 1: 1%–5%; 2: 5%–10%; 3: 10%–20%; 4: >20% loss of weight; and 5: no survival); (2) stool consistency (0: normal stools; 1: loose stools; 2: watery diarrhea; 3: watery diarrhea with blood; and 4: no survival); and (3) the general activity (0: normal; 1–2: moderate activity; 3: null activity; and 4: no survival). Fold change in body weights was calculated as the difference from the body weights on day 0 at the starting of DSS treatment and the body weights at the different days along the experiment, expressed as a percentage.

Peripheral blood samples were analyzed using an automated blood cell counter (Sysmex analyzer, XN‐1000 Pure) at various time points.

### 4.4 Histology analysis

Colons were surgically removed and fixed in formalin overnight. Colon tissue segments were collected, embedded in paraffin, and stained with hematoxylin and eosin. Microscopic examinations were performed to assess the presence of infiltrating mononuclear cells and structural integrity.

### 4.5 Ex vivo imaging and analysis

For labelling WT and CXCR4‐IL10‐MSCs, Ad‐MSCs were incubated with XenoLight DiR (DiIC18 (7) or 1,1′‐dioctadecyltetramethyl indotricarbocyanine Iodide, 1.6 μg/mL, Perkin Elmer, MA, USA) for 3 min at room temperature and then centrifuged twice with phosphate‐buffered saline (PBS) for 5 min at 1400 rpm. DiR‐labeled MSCs were resuspended in Ringer's lactate and 3–5 × 10^6^ cells/200 μL were intraperitoneally injected within 1 h after labelling in healthy mice and on day 5 of the 7‐day DSS cycle in colitic mice. Mice were sacrificed at 2, 24, 48, 120, 168 and 192 h post‐MSC infusion to collect caudal and mesenteric lymph nodes, blood, bone marrow (one femur and one tibia), colon, heart, kidneys, liver, lungs, peritoneal cavity serous membrane and fluid, reproductive organs, small intestine, spleen, stomach and thymus for ex vivo imaging. Bone marrow cells from one femur and one tibia were flushed out with 2 mL of PBS. Cells within the peritoneal cavity were collected with 1 mL of PBS. The collected organs, along with 50 μL of blood, bone marrow and peritoneal lavages were imaged using the IVIS LUMINA XRM. Filter conditions and illumination settings for DiR imaging were set at an excitation/emission 710/760 nm, high lamp level, binning 4 and automatic exposure time. Fluorescent images of each organ were analyzed using Living Image software. Regions of interests (ROIs) of each organ were drawn over the organ images and the distribution of each DiR‐labeled MSCs in each organ was quantified as total radiant efficiency ([p/s]/[μW/cm^2^]). Total radiance efficiency in blood was calculated after scaling to 2 mL of blood in the whole mouse body. Tissue values of total radiant efficiency below 1.6 × 10^9^ [p/s]/[μW/cm^2^] and blood values below 1.3 × 10^10^ [p/s]/[μW/cm^2^] (10% of total signal) were considered negative, based on the total radiant efficiency of untreated mice. Maximum total radiant efficiency (100%) was calculated by the sum of the total radiant efficiency in the tissues and organs analyzed at 2 h, along with the total bioluminescence signal of DiR‐labeled MSCs before injection.

### 4.6 Flow cytometry analysis

Mice were culled and different tissues and organs like bone marrow, colon, liver, peritoneal cavity lavage, peripheral blood and spleen were collected. Bone marrow cells and cells from peritoneal cavity were isolated. Colon leukocytes were isolated following the manufacturer's instructions for the Lamina Propria Dissociation Kit (Miltenyi Biotech, Germany). Isolated mononuclear cells were surface‐stained with antibodies listed in Table S1. For intracellular staining, Forkhead box P3 (Foxp3)/Transcription factor staining buffer set was used according to the manufacturer's instructions (ThermoFisher Scientific, MA, USA). For the intracellular analysis of arginase (Arg) 1, inducible nitric oxide synthase (iNOS) and cytokine expression, mononuclear cells were stimulated overnight with 5 ng/mL phorbol myristate acetate, 500 ng/mL ionomycin and/or 0.1 μg/mL lipopolysaccharide, in the presence of TNF alpha protease inhibitor I (TAPI‐1), GolgiPlug and GolgiStop (BD Biosciences) for cytokine expression. Cells were fixed and intracellularly stained with antibodies described in Table S1. Cells were collected on a BD LSR Fortessa flow cytometer using DAPI to analyze non‐viable cells.

For multiparametric analyses, dimensional reduction and clustering were performed using OMIQ data analysis software (OMIQ, Inc. Santa Clara, CA). CD45‐positive events from all samples were selected for subsequent analysis on the OMIQ platform. The FlowAI algorithm was employed to check and exclude any aberrant regions from all analyzed files. Subsequently, a *t*‐SNE analysis was performed to visualize the different CD45, CD11b and CD4^+^CD3^+^ subsets across groups. FlowSOM was utilized to cluster the data through metaclustering with all clusters plotted on traditional dot plots for phenotypic confirmation following standard manual gating analysis.

### 4.7 Quantitative RT‐PCR

Total RNA was extracted from colon tissues using RNeasy® Mini Kit (Qiagen, Germany). cDNA was synthesized from total RNA using the SuperScript™ VILO™ MasterMix (ThermoFisher, MA, USA) according to the manufacturer's instructions. Quantitative PCR was performed using SYBR Green Master Mix (Applied Biosystem, MA, USA). mRNA expression levels were normalized to GAPDH and healthy mice mRNA levels, which was calculated using the 2−^ΔΔ^Ct method. Primer sequences for qRT‐PCR are listed in Table S2.

### 4.8 Statistical analysis

Normal distribution was assessed using the Shapiro‐Wilks test. For normally distributed data, parametric tests (*t* Student test) were applied, while non‐parametric tests (*U* Mann–Whitney) were used for non‐normally distributed data. The log rank test was utilized for survival analysis and Fisher's exact test was employed for contingency analysis. Statistical analysis was performed using GraphPad Prism 10.2 software.

## 5 CONCLUSIONS

MSCs possess profound immunomodulatory and regenerative properties, making them attractive for use in cellular and regenerative therapies. Although hundreds of clinical trials are ongoing to test their utility as a cellular therapy for acute and chronic degenerative and inflammatory disorders, MSC‐based therapy is currently exhibiting considerable uncertainty due to contradictory clinical efficacy. Among others, variations in MSC products and clinical indications being tested, as well as insufficient clinical potency assessment, are currently limiting their successful adoption in the clinical arena. Hence, among the scientific community, a general consensus exists on the need to develop next‐generation MSC‐based therapies aimed at achieving more consistent and sustainable clinical efficacy.

In this study, we demonstrate that genetically engineered adipose tissue‐derived human MSCs, constitutively expressing the CXC chemokine receptor 4 (CXCR4) and interleukin 10 (IL10) (CXCR4‐IL10‐MSCs), exhibit enhanced therapeutic effects compared to wild‐type MSCs in a preclinical model of DSS‐induced ulcerative colitis. Strikingly, we also observed long‐term protective immune memory effects in response to a recurrent inflammatory challenge. Overall, we demonstrate that the proposed strategy, based on enhancing the homing and the immunosuppressive abilities of MSCs, represents an optimized MSC‐based cell product for refractory IBD where complete resolution of the inflammation is impaired.

## AUTHOR CONTRIBUTIONS

Conceptualization: M.L‐S., J.A.B., R.M.Y., and M.I.G.; Data curation: M.L‐S., M.C.O‐V., M.F‐G., M.H‐R., and R.M.Y.; Funding Acquisition: J.A.B. and M.I.G.; Investigation and Methodology: M.L‐S., M.C.O‐V., R.M.Y., and M.I.G.; Writing original draft: M.L‐S., J.A.B., and M.I.G.; Writing—review and editing: M.L‐S., M.C.O‐V., M.F.G., M.H‐R., J.A.B., R.M.Y., and M.I.G.

## FUNDING INFORMATION

This work was supported by Instituto de Salud Carlos III (ISCIII) [grant numbers PI21/01441, RICORS‐RD21/0017/0027 and PIE15/00048]; and the Comunidad Autónoma de Madrid [grant number, B2017/BMD3692].

## CONFLICT OF INTEREST STATEMENT

Maria Fernandez‐Garcia, Juan Antonio Bueren Roncero, Rosa Maria Yañez, Mercedes Lopez‐Santalla, and Marina Inmaculada Garin are co‐founders of Kiji Therapeutics. M.L.S., M.F.G., M.H.R., J.A.B., R.M.Y., and M.I.G. are co‐inventors of a related patent (PCT/EP2021/074612).

## Supporting information


**FIGURE S1:** Analysis of total radiance efficiency in healthy and DSS‐induced colitic mice following intraperitoneal infusion of Dir‐labeled WT‐ or CXCR4‐IL10‐MSCs in various tissues and organs. (a) Total radiant efficiency ([p/s]/[μW/cm^2^]) in healthy mice (triangles) and DSS‐induced colitis mice (circles) over 168 h following intraperitoneal injection of Dir‐labeled WT‐ (green) or CXCR4‐IL10‐ MSCs (blue). Total radiant efficiency ([p/s]/[μW/cm^2^]) was measured in the liver, blood, small intestine, stomach, spleen, peritoneal cavity lavage, bone marrow (one paw) lavage, colon, peritoneal serous membrane, lungs and kidneys 2 h post‐administration of WT‐ (green) and CXCR4‐IL10 (blue) Dir‐labeled MSCs in healthy mice (b) and 2 h (c) and 48 h (d) post‐injection in DSS‐induced colitic mice. Healthy + Dir‐labeled WT‐MSCs (green triangle, *n* = 3–6), Healthy + Dir‐labeled CXCR4‐IL10‐MSCs (blue triangle, *n* = 3–8), DSS‐induced colitic mice + Dir‐labeled WT‐MSCs (green circles, *n* = 4–12) and DSS‐induced colitic mice + Dir‐labeled CXCR4‐IL10‐MSCs (green circles, *n* = 4–14). Data are presented as the mean and interquartile range (p75, upper edge; p25, lower edge; p50, midline) of total radiant efficiency ([p/s]/[μW/cm^2^]). Statistical significance was analyzed using the Mann–Whitney *U* test, with *p* = 0.1–0.05 indicated by numbers and ^#^ *p* < 0.05 denoting significance. Results represent seven independent experiments. (E) Representative images showing regions of interest (ROI) in the kidneys and peritoneal serious membrane (row 1), caudal and mesenteric lymph nodes and colon (row 2), stomach and small intestine (row 3), spleen and liver (row 4), thymus, lungs and heart (row 5), female reproductive organs (row 6) and bone marrow and peritoneal cavity lavages (row 7) depicting total radiant efficiency ([p/s]/[μW/cm^2^]) for three representative mice 24 h post‐injection of Dir‐labeled WT‐ (left) or CXCR4‐IL10‐ MSCs (right).

**FIGURE S2:** Characterization of the immune system in peripheral blood and colon of mice during the 1st 7‐day DSS cycle following intraperitoneal injection of WT‐ and CXCR4‐IL10‐MSCs on day 5. White blood cell counts (a), granulocytes (b), monocytes (c), lymphocytes (d), platelets (e), red blood cells (f) (in 10^6^/mL) and hemoglobin (g) (in g/dL) in healthy (black, *n* = 42), WT‐ (green, *n* = 10) and CXCR4‐IL10‐MSC‐treated (blue, *n* = 10) and untreated (red, *n* = 10) colitic mice during the first 7‐day DSS cycle. Intracellular cytokine expression within CD45^+^ cells on day 6 of the 7‐day DSS cycle (h). Violin plots display median and interquartile range (p75, upper edge; p25, lower edge; p50, midline) for mRNA levels of IFNγ (i), TNFα (j), IL6 (k), cyclooxygenase 2 (COX‐2, l), inducible nitric oxide synthase (iNOS, m) and transforming growth factor (TGFβ, n) in the colon, measured by qRT‐PCR on day 8 after 7 day‐DSS cycle in healthy (white, *n* = 20), WT‐ (green, *n* = 12) and CXCR4‐IL10‐MSC‐treated (blue, *n* = 19) and untreated (red, *n* = 9) colitic mice. Percentages of T cell subtypes (CD4^+^CD3^+^ or CD8^+^CD3^+^) measured by flow cytometry from day 6 to day 13 in the colon of healthy, WT‐ and CXCR4‐IL10‐MSC‐treated and untreated colitic mice (o). Statistical significance was determined by Mann–Whitney *U* test and represented by *p < 0.05, **p < 0.01, ***p < 0.001 and ****p ≤ 0.0001 DSS versus heathy; ^+^ *p* < 0.05, ^++^ *p* < 0.01, ^+++^ *p* < 0.001 and ^++++^ *p* ≤ 0.0001 DSS + WT‐MSCs versus healthy; ^%^ *p* < 0.05, ^%%^ *p* < 0.01, ^%%%^ *p* < 0.001 and ^%%%%^ *p* ≤ 0.0001 DSS + CXCR4‐IL10‐MSCs versus healthy; ^$^ *p* < 0.05 DSS + CXCR4‐IL10‐MSCs versus DSS and ^&^ *p* < 0.05 DSS + CXCR4‐IL10‐MSCs versus DSS + WT‐MSCs. Results correspond to seven independent experiments.

**FIGURE S3:** *t*‐Distributed stochastic neighbor embedding (*t*‐SNE) projection of CD11b^+^ myeloid cells. Clustering of CD11b^+^ cells using *t*‐SNE plots, identifying myeloid cell populations. Relevant phenotypic marker expression (CD45, CD11b, Ly6G, Ly6C, F4/80, and MHC‐II) overlaid onto the t‐SNE map on days 7, 10 and 13. Healthy (*n* = 6), DSS (*n* = 13), DSS + WT‐MSCs (*n* = 12), and DSS + CXCR4‐IL10‐MSCs (*n* = 11).

**FIGURE S4:** *t*‐Distributed stochastic neighbor embedding (*t*‐SNE) projection of CD4^+^CD3^+^ lymphoid cells. Clustering of CD4^+^CD3^+^ cells using *t*‐SNE plots identifying FOXP3^+^CD4^+^ T cells. Relevant phenotypic marker expression (CD45, CD3, CD4, and FOXP3) overlaid onto the *t*‐SNE map on day 13. Healthy (*n* = 2), DSS (*n* = 6), DSS + WT‐MSCs (*n* = 6) and DSS + CXCR4‐IL10‐MSCs (*n* = 6).

**FIGURE S5:** Peripheral blood and colon status of healthy, WT‐ and CXCR4‐IL10‐MSC‐treated and untreated colitic mice 3 months after the first 7‐day DSS cycle. White blood cell counts (a), granulocytes (b), monocytes (c), lymphocytes (d), platelets (e), red blood cells (f) (×10^6^/mL) and hemoglobin (g, g/dL) of healthy (black), WT‐ (green) and CXCR4‐IL10‐MSC‐treated (blue) and untreated (red) colitic mice on day 30, 60 and 90 post‐7‐day DSS cycle. Body weights (g) (H), representative colon images at 50× (left) and 200× (right) magnification (i), number (×10^6^, j) and percentage of CD45^+^ (k) on day 90 post‐7‐day DSS cycle in healthy (*n* = 53), WT‐ (*n* = 4–8) and CXCR4‐IL10‐MSC (*n* = 7–10)‐treated and untreated (*n* = 4–8) colitic mice. Data are presented as interquartile ranges (p75, upper edge; p25, lower edge; p50, midline) for hematological data and mean ± standard error of the mean for cell numbers. Statistical significance was determined by Mann–Whitney *U* test (**p < 0.01 DSS vs. heathy; ^++^ *p* < 0.01 DSS + WT‐MSCs vs. healthy and ^%%^ *p* < 0.01 DSS + CXCR4‐IL10‐MSCs vs. healthy). Results represent two independent experiments.

**FIGURE S6:** Immune responses in the colon of healthy, untreated and WT‐ and CXCR4‐IL10‐ MSC‐treated colitic mice on day 7 during the 2nd 7‐day DSS cycle following a 12‐week latency period. B220^+^ (a), Gr1^int^CD11b^+^ (b) and PDL1^+^ (within F4/80^+^, c) cells (in percentage, %). Representative histograms for PDL1 expression within F4/80^+^ cells (d) on day 7 of the 2nd challenge of 7‐day DSS cycle in the colon of healthy (*n* = 4–8), untreated (*n* = 4–7), WT‐ (*n* = 4–7) and CXCR4‐IL10‐ (*n* = 3–6) MSC‐treated colitic mice. Violin plots show medians and interquartile ranges (p75, upper edge; p25, lower edge; p50, midline) for mRNA levels of IFNγ (e), TNFα (f), IL6 (g), cyclooxygenase 2 (COX‐2, h) and inducible nitric oxide synthase (iNOS, i) measured by qRT‐PCR on day 8 of the second 7‐day DSS cycle in colon of healthy (white, *n* = 4–11) and WT‐ (green, *n* = 10–12) and CXCR4‐IL10‐MSC‐treated (blue, *n* = 19) and untreated (red, *n* = 10–12) colitic mice. Significance was analyzed by the Mann–Whitney *U* test and represented by number if *p* = 0.1–0.05, **p < 0.01, ***p < 0.001 and ****p < 0.0001 DSS versus heathy; ^+++^ *p* < 0.001 and ^++++^ *p* < 0.0001 DSS + WT‐MSCs versus healthy, ^%%%^ *p* < 0.001 and ^%%%%^ *p* < 0.0001 DSS + CXCR4‐IL10‐MSCs versus healthy, ^$^ *p* < 0.05 DSS + CXCR4‐IL10‐MSCs versus DSS, ^&^ *p* < 0.05 DSS + WT‐MSCs versus DSS and ^#^ *p* < 0.05 and ^##^ *p* < 0.01 DSS + CXCR4/IL10‐MSCs versus DSS + WT‐MSCs. Results correspond to two independent experiments.

**FIGURE S7:** Schematic map of the bicistronic lentiviral vector PGK‐CXCR4‐IL10. The map illustrates the main functional elements of the backbone and expression cassette, including the 5′ and 3′ self‐inactivating LTRs, packaging signal (Ψ), central polypurine tract (cPPT/CTS), Rev‐responsive element (RRE), PGK promoter, and a bicistronic transgene consisting of codon‐optimized CXCR4 and IL10 sequences linked by the self‐cleaving peptide E2A. Additional elements include the mutated woodchuck hepatitis virus post‐transcriptional regulatory element (WPRE*) and the polyadenylation signal (polyA).

**TABLE S1:** List of antibodies used for flow cytometry.

**TABLE S2:** List of primers used for mouse.

## Data Availability

The data that support the findings of this study are available from the corresponding author upon reasonable request.
