# Supplementary figures and images for: Improved efficacy and long‐term protective effects of CXCR4/IL10 bioengineered mesenchymal stromal cells in a model of inflammatory bowel disease
Source: Bioeng Transl Med. 2025 Dec 16;11(2):e70083. doi: 10.1002/btm2.70083 (PMC13093540; doi:10.1002/btm2.70083)

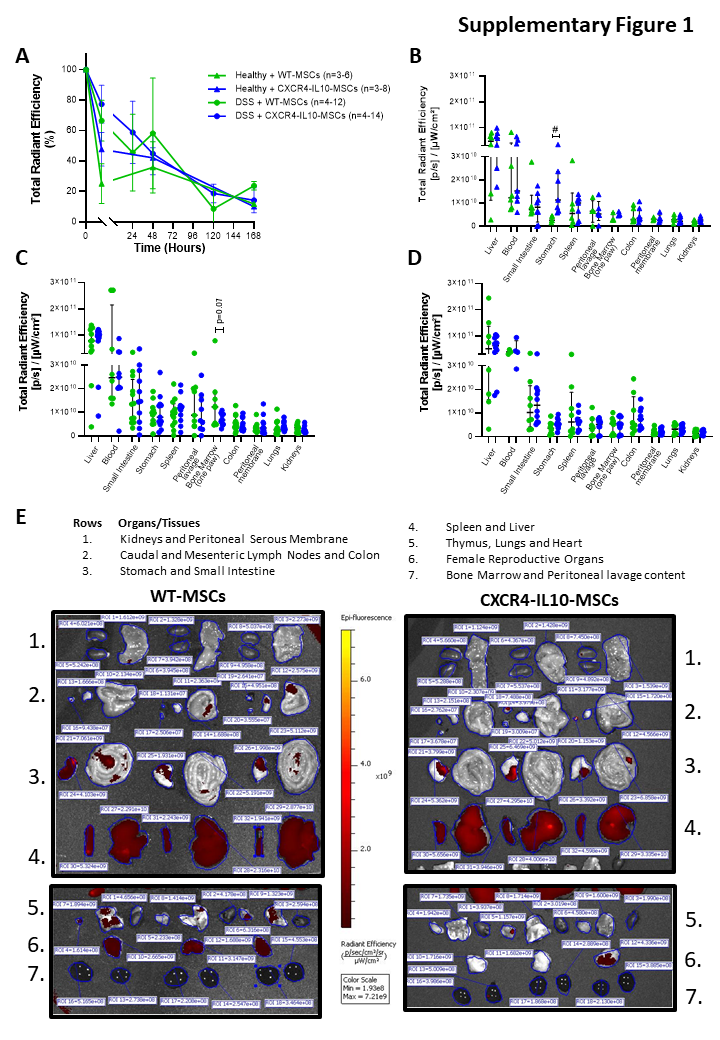

Supplement: Supplementary file 1 — FIGURE S1: Analysis of total radiance efficiency in healthy and DSS‐induced colitic mice following intraperitoneal infusion of Dir‐labeled WT‐ or CXCR4‐IL10‐MSCs in various tissues and organs. (a) Total radiant efficiency ([p/s]/[μW/cm2]) in healthy mice (triangles) and DSS‐induced colitis mice (circles) over 168 h following intraperitoneal injection of Dir‐labeled WT‐ (green) or CXCR4‐IL10‐ MSCs (blue). Total radiant efficiency ([p/s]/[μW/cm2]) was measured in the liver, blood, small intestine, stomach, spleen, peritoneal cavity lavage, bone marrow (one paw) lavage, colon, peritoneal serous membrane, lungs and kidneys 2 h post‐administration of WT‐ (green) and CXCR4‐IL10 (blue) Dir‐labeled MSCs in healthy mice (b) and 2 h (c) and 48 h (d) post‐injection in DSS‐induced colitic mice. Healthy + Dir‐labeled WT‐MSCs (green triangle, n = 3–6), Healthy + Dir‐labeled CXCR4‐IL10‐MSCs (blue triangle, n = 3–8), DSS‐induced colitic mice + Dir‐labeled WT‐MSCs (green circles, n = 4–12) and DSS‐induced colitic mice + Dir‐labeled CXCR4‐IL10‐MSCs (green circles, n = 4–14). Data are presented as the mean and interquartile range (p75, upper edge; p25, lower edge; p50, midline) of total radiant efficiency ([p/s]/[μW/cm2]). Statistical significance was analyzed using the Mann–Whitney U test, with p = 0.1–0.05 indicated by numbers and # p < 0.05 denoting significance. Results represent seven independent experiments. (E) Representative images showing regions of interest (ROI) in the kidneys and peritoneal serious membrane (row 1), caudal and mesenteric lymph nodes and colon (row 2), stomach and small intestine (row 3), spleen and liver (row 4), thymus, lungs and heart (row 5), female reproductive organs (row 6) and bone marrow and peritoneal cavity lavages (row 7) depicting total radiant efficiency ([p/s]/[μW/cm2]) for three representative mice 24 h post‐injection of Dir‐labeled WT‐ (left) or CXCR4‐IL10‐ MSCs (right). [file BTM2-11-e70083-s003.tif]

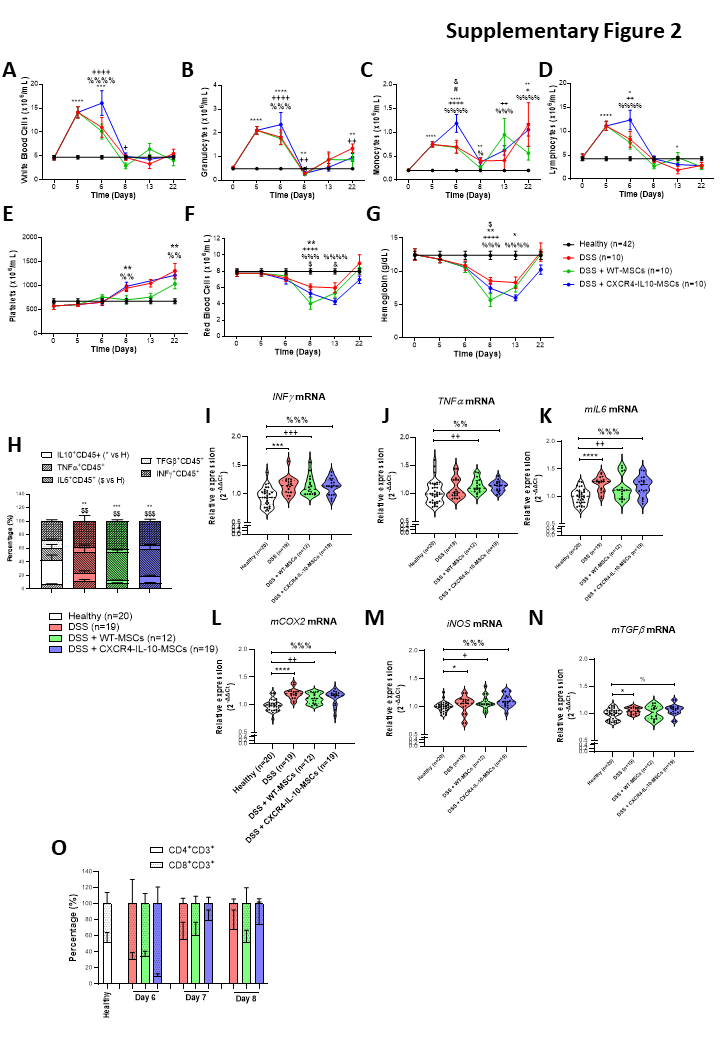

Supplement: Supplementary file 2 — FIGURE S2: Characterization of the immune system in peripheral blood and colon of mice during the 1st 7‐day DSS cycle following intraperitoneal injection of WT‐ and CXCR4‐IL10‐MSCs on day 5. White blood cell counts (a), granulocytes (b), monocytes (c), lymphocytes (d), platelets (e), red blood cells (f) (in 106/mL) and hemoglobin (g) (in g/dL) in healthy (black, n = 42), WT‐ (green, n = 10) and CXCR4‐IL10‐MSC‐treated (blue, n = 10) and untreated (red, n = 10) colitic mice during the first 7‐day DSS cycle. Intracellular cytokine expression within CD45+ cells on day 6 of the 7‐day DSS cycle (h). Violin plots display median and interquartile range (p75, upper edge; p25, lower edge; p50, midline) for mRNA levels of IFNγ (i), TNFα (j), IL6 (k), cyclooxygenase 2 (COX‐2, l), inducible nitric oxide synthase (iNOS, m) and transforming growth factor (TGFβ, n) in the colon, measured by qRT‐PCR on day 8 after 7 day‐DSS cycle in healthy (white, n = 20), WT‐ (green, n = 12) and CXCR4‐IL10‐MSC‐treated (blue, n = 19) and untreated (red, n = 9) colitic mice. Percentages of T cell subtypes (CD4+CD3+ or CD8+CD3+) measured by flow cytometry from day 6 to day 13 in the colon of healthy, WT‐ and CXCR4‐IL10‐MSC‐treated and untreated colitic mice (o). Statistical significance was determined by Mann–Whitney U test and represented by *p < 0.05, **p < 0.01, ***p < 0.001 and ****p ≤ 0.0001 DSS versus heathy; + p < 0.05, ++ p < 0.01, +++ p < 0.001 and ++++ p ≤ 0.0001 DSS + WT‐MSCs versus healthy; % p < 0.05, %% p < 0.01, %%% p < 0.001 and %%%% p ≤ 0.0001 DSS + CXCR4‐IL10‐MSCs versus healthy; $ p < 0.05 DSS + CXCR4‐IL10‐MSCs versus DSS and & p < 0.05 DSS + CXCR4‐IL10‐MSCs versus DSS + WT‐MSCs. Results correspond to seven independent experiments. [file BTM2-11-e70083-s009.tif]

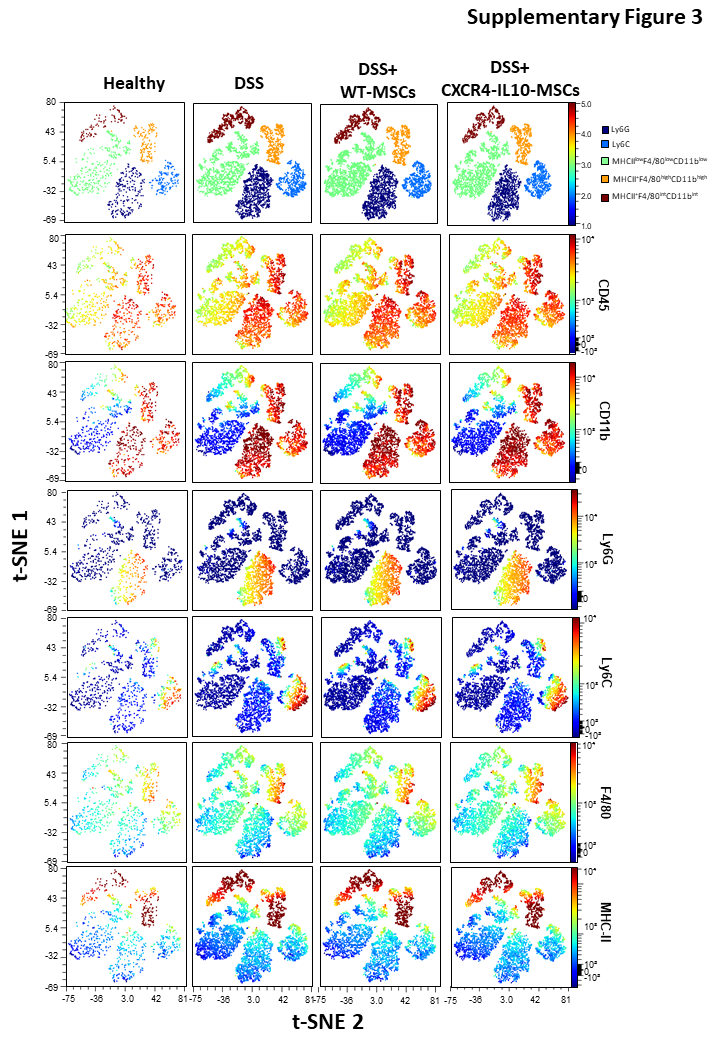

Supplement: Supplementary file 3 — FIGURE S3: t‐Distributed stochastic neighbor embedding (t‐SNE) projection of CD11b+ myeloid cells. Clustering of CD11b+ cells using t‐SNE plots, identifying myeloid cell populations. Relevant phenotypic marker expression (CD45, CD11b, Ly6G, Ly6C, F4/80, and MHC‐II) overlaid onto the t‐SNE map on days 7, 10 and 13. Healthy (n = 6), DSS (n = 13), DSS + WT‐MSCs (n = 12), and DSS + CXCR4‐IL10‐MSCs (n = 11). [file BTM2-11-e70083-s005.tif]

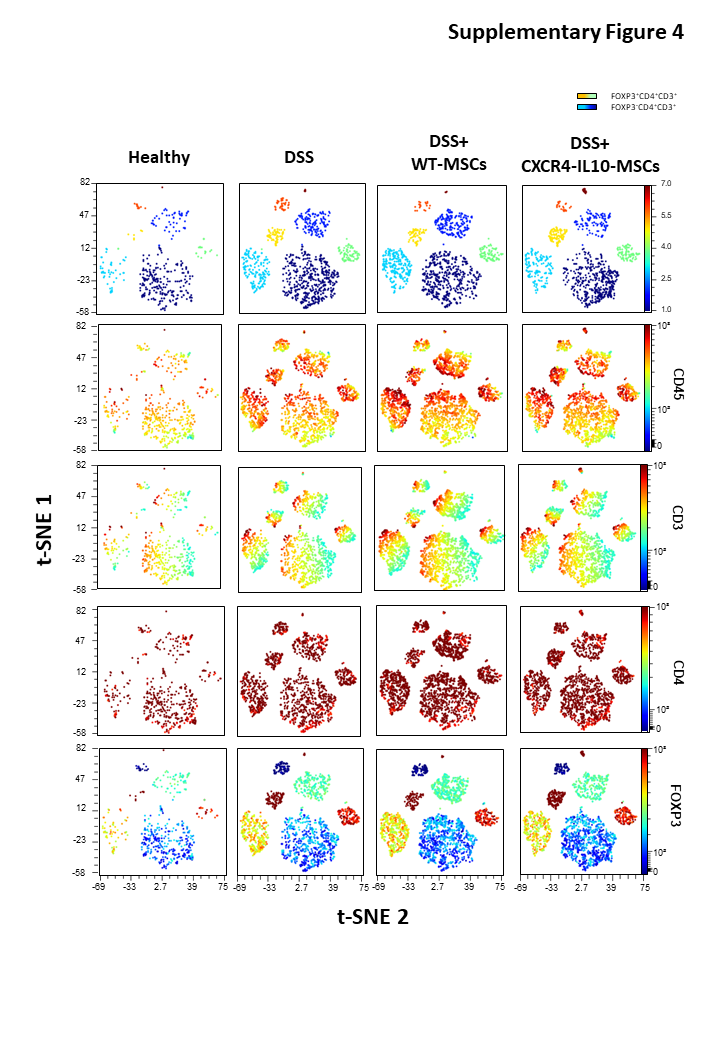

Supplement: Supplementary file 4 — FIGURE S4: t‐Distributed stochastic neighbor embedding (t‐SNE) projection of CD4+CD3+ lymphoid cells. Clustering of CD4+CD3+ cells using t‐SNE plots identifying FOXP3+CD4+ T cells. Relevant phenotypic marker expression (CD45, CD3, CD4, and FOXP3) overlaid onto the t‐SNE map on day 13. Healthy (n = 2), DSS (n = 6), DSS + WT‐MSCs (n = 6) and DSS + CXCR4‐IL10‐MSCs (n = 6). [file BTM2-11-e70083-s001.tif]

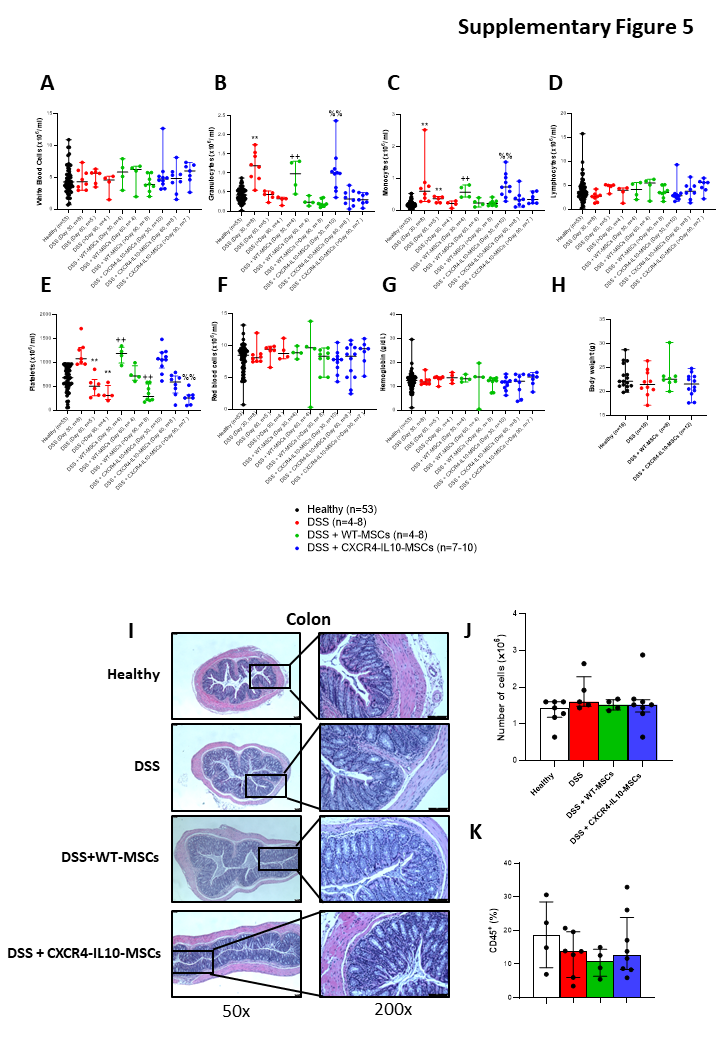

Supplement: Supplementary file 5 — FIGURE S5: Peripheral blood and colon status of healthy, WT‐ and CXCR4‐IL10‐MSC‐treated and untreated colitic mice 3 months after the first 7‐day DSS cycle. White blood cell counts (a), granulocytes (b), monocytes (c), lymphocytes (d), platelets (e), red blood cells (f) (×106/mL) and hemoglobin (g, g/dL) of healthy (black), WT‐ (green) and CXCR4‐IL10‐MSC‐treated (blue) and untreated (red) colitic mice on day 30, 60 and 90 post‐7‐day DSS cycle. Body weights (g) (H), representative colon images at 50× (left) and 200× (right) magnification (i), number (×106, j) and percentage of CD45+ (k) on day 90 post‐7‐day DSS cycle in healthy (n = 53), WT‐ (n = 4–8) and CXCR4‐IL10‐MSC (n = 7–10)‐treated and untreated (n = 4–8) colitic mice. Data are presented as interquartile ranges (p75, upper edge; p25, lower edge; p50, midline) for hematological data and mean ± standard error of the mean for cell numbers. Statistical significance was determined by Mann–Whitney U test (**p < 0.01 DSS vs. heathy; ++ p < 0.01 DSS + WT‐MSCs vs. healthy and %% p < 0.01 DSS + CXCR4‐IL10‐MSCs vs. healthy). Results represent two independent experiments. [file BTM2-11-e70083-s008.tif]

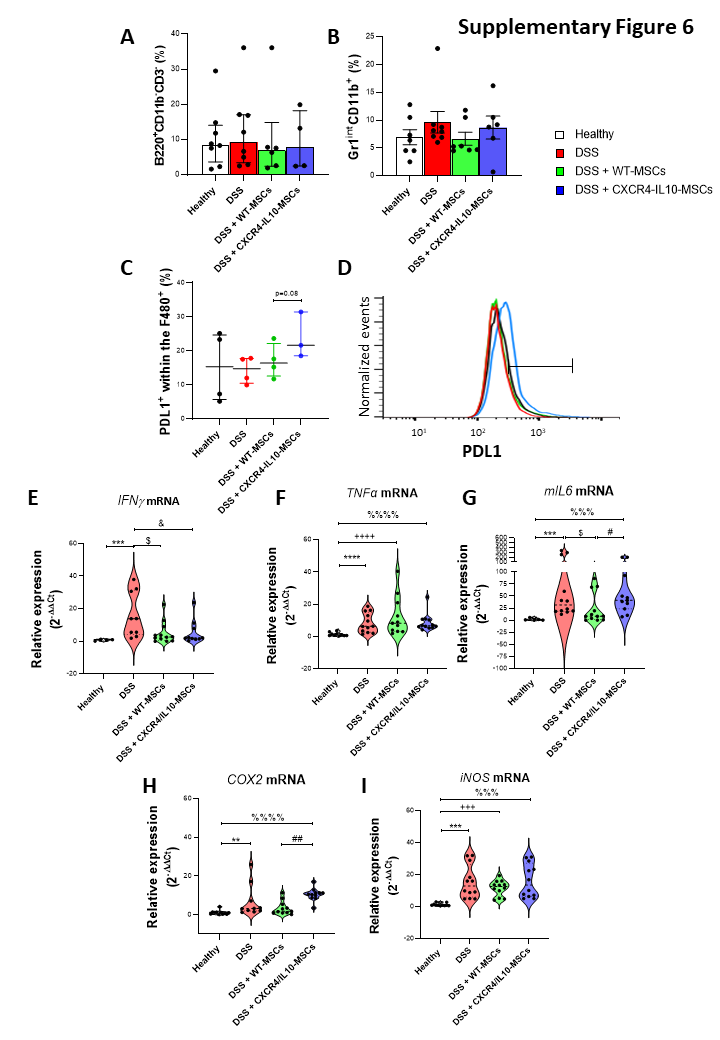

Supplement: Supplementary file 6 — FIGURE S6: Immune responses in the colon of healthy, untreated and WT‐ and CXCR4‐IL10‐ MSC‐treated colitic mice on day 7 during the 2nd 7‐day DSS cycle following a 12‐week latency period. B220+ (a), Gr1intCD11b+ (b) and PDL1+ (within F4/80+, c) cells (in percentage, %). Representative histograms for PDL1 expression within F4/80+ cells (d) on day 7 of the 2nd challenge of 7‐day DSS cycle in the colon of healthy (n = 4–8), untreated (n = 4–7), WT‐ (n = 4–7) and CXCR4‐IL10‐ (n = 3–6) MSC‐treated colitic mice. Violin plots show medians and interquartile ranges (p75, upper edge; p25, lower edge; p50, midline) for mRNA levels of IFNγ (e), TNFα (f), IL6 (g), cyclooxygenase 2 (COX‐2, h) and inducible nitric oxide synthase (iNOS, i) measured by qRT‐PCR on day 8 of the second 7‐day DSS cycle in colon of healthy (white, n = 4–11) and WT‐ (green, n = 10–12) and CXCR4‐IL10‐MSC‐treated (blue, n = 19) and untreated (red, n = 10–12) colitic mice. Significance was analyzed by the Mann–Whitney U test and represented by number if p = 0.1–0.05, **p < 0.01, ***p < 0.001 and ****p < 0.0001 DSS versus heathy; +++ p < 0.001 and ++++ p < 0.0001 DSS + WT‐MSCs versus healthy, %%% p < 0.001 and %%%% p < 0.0001 DSS + CXCR4‐IL10‐MSCs versus healthy, $ p < 0.05 DSS + CXCR4‐IL10‐MSCs versus DSS, & p < 0.05 DSS + WT‐MSCs versus DSS and # p < 0.05 and ## p < 0.01 DSS + CXCR4/IL10‐MSCs versus DSS + WT‐MSCs. Results correspond to two independent experiments. [file BTM2-11-e70083-s006.tif]

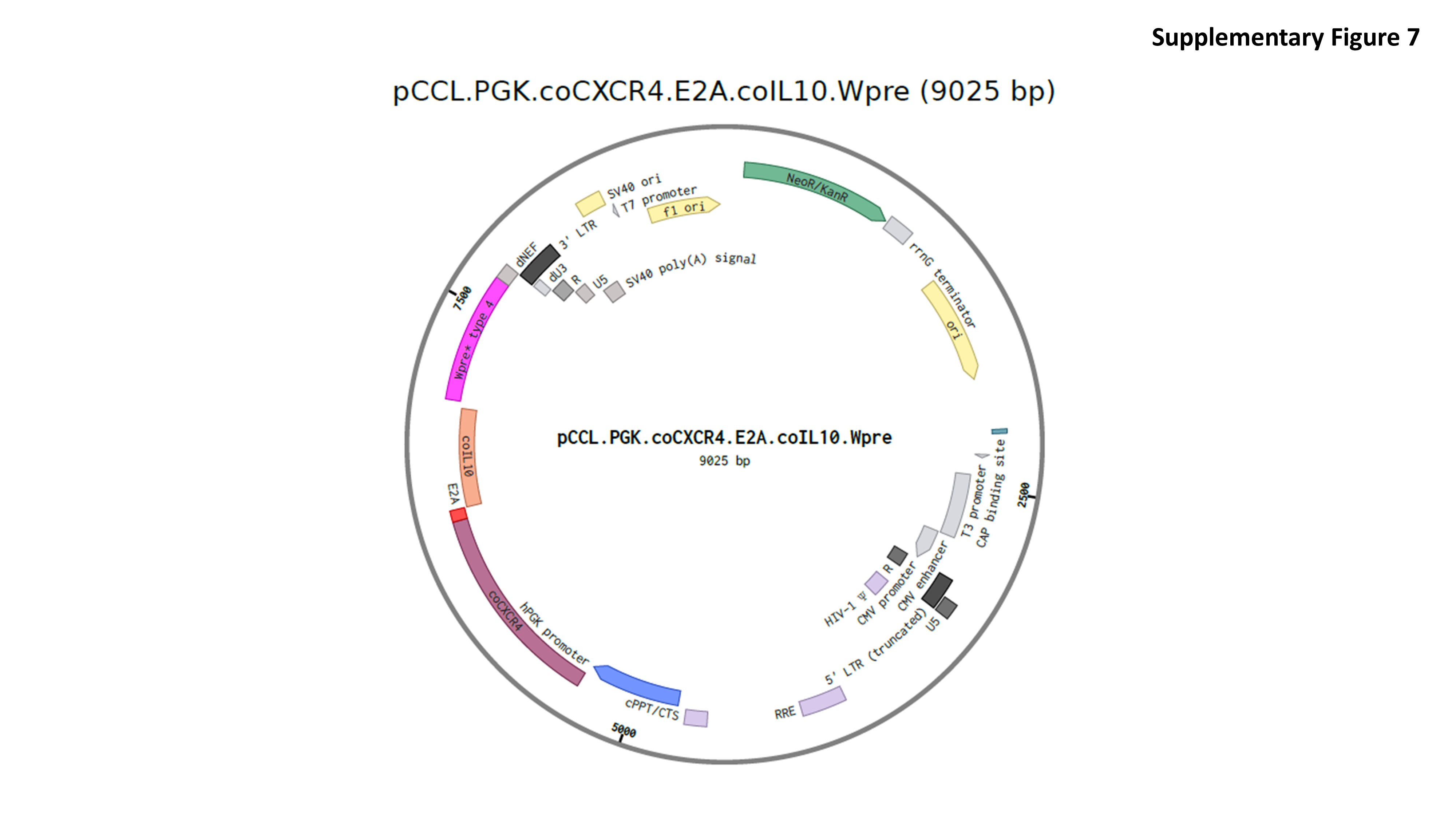

Supplement: Supplementary file 7 — FIGURE S7: Schematic map of the bicistronic lentiviral vector PGK‐CXCR4‐IL10. The map illustrates the main functional elements of the backbone and expression cassette, including the 5′ and 3′ self‐inactivating LTRs, packaging signal (Ψ), central polypurine tract (cPPT/CTS), Rev‐responsive element (RRE), PGK promoter, and a bicistronic transgene consisting of codon‐optimized CXCR4 and IL10 sequences linked by the self‐cleaving peptide E2A. Additional elements include the mutated woodchuck hepatitis virus post‐transcriptional regulatory element (WPRE*) and the polyadenylation signal (polyA). [file BTM2-11-e70083-s002.tif]
