# Supplementary material for: Improved efficacy and long‐term protective effects of CXCR4/IL10 bioengineered mesenchymal stromal cells in a model of inflammatory bowel disease
Source: Bioeng Transl Med. 2025 Dec 16;11(2):e70083. doi: 10.1002/btm2.70083 (PMC13093540; doi:10.1002/btm2.70083)
Supplement: Supplementary file 8 — TABLE S1: List of antibodies used for flow cytometry. [file BTM2-11-e70083-s007.docx]

**Supplementary Table 1**. List of antibodies used for Flow Cytometry

| **Antibody** | **Clone** | **Company** | **City, Country** |
| --- | --- | --- | --- |
| Arginase-1 (Arg-1) | A1exF5 | eBiosciences | Waltham, MA USA |
| B220 | RA3-6B2 | Biolegend/Becton Dickinson | San Diego, CA, USA/ Franklin Lakes, NJ, USA |
| CCR2 | SA203G11 | Biolegend | San Diego, CA, USA |
| CD3 | 145-2C11 | Becton Dickinson | Franklin Lakes, NJ, USA |
|  |  | Miltenyi Biotech | Bergisch Gladbach, Germany |
| CD4 | RM4-5 | Biolegend | San Diego, CA, USA |
| CD11b | M1/70 | eBiosciences | Waltham, MA USA |
|  |  | Becton Dickinson | Franklin Lakes, NJ, USA |
| CD11c | HL3 | Becton Dickinson | Franklin Lakes, NJ, USA |
| CD45 | 30F11 | Becton Dickinson | Franklin Lakes, NJ, USA |
|  |  | Miltenyi Biotech | Bergisch Gladbach, Germany |
| CD73 | TY/11.8 | eBiosciences | Waltham, MA USA |
| CD80 | 16-10A1 | Becton Dickinson | Franklin Lakes, NJ, USA |
| CD127 | SB/199 | Becton Dickinson | Franklin Lakes, NJ, USA |
| CD163 | S15049I | Biolegend | San Diego, CA, USA |
| CD169 | 3D6.112 | Biolegend | San Diego, CA, USA |
| CD206 | MR5D3 | Becton Dickinson | Franklin Lakes, NJ, USA |
| CX3CR1 | Q9Z0D | RyD systems | [Minneapolis,](https://www.google.com/search?sxsrf=ACYBGNRXwm2iatQyZZqmWzsB4oeZqBzakg:1579260680721&q=Minneapolis&stick=H4sIAAAAAAAAAOPgE-LSz9U3MMmurEwrUOIEsdMKqsrTtLSyk63084vSE_MyqxJLMvPzUDhWGamJKYWliUUlqUXFi1i5fTPz8lITC_JzMot3sDICAH3iWQ1WAAAA&sa=X&ved=2ahUKEwii-oa_xIrnAhVQCxoKHRwvAgQQmxMoATAQegQICRAE&sxsrf=ACYBGNRXwm2iatQyZZqmWzsB4oeZqBzakg:1579260680721) MN, USA |
| F4/80 | BM8 | eBiosciences | Waltham, MA USA |
| FOXP3 | FJK-16s | eBiosciences | Waltham, MA USA |
| GM-CSF | MP1-22E9 | eBiosciences | Waltham, MA USA |
| Gr1 | RB6-8C5 | Biolegend/Becton Dickinson | San Diego, CA, USA/ Franklin Lakes, NJ, USA |
| Inducible nitric oxide synthase (iNOS) | CXNFT | Invitrogen | Waltham, MA USA |
| Interferon γ (IFN- γ) | XMG1.2 | eBiosciences | Waltham, MA USA |
| Interleukin-6 (IL-6) | MP5-20F3 | Biolegend | San Diego, CA, USA |
| Interleukin-10 (IL-10) | JES5-16E3 | Becton Dickinson | Franklin Lakes, NJ, USA |
| Ly6C | 1G7.G10 | Miltenyi Biotech | Bergisch Gladbach, Germany |
| Ly6G | 1A8 | Miltenyi Biotech | Bergisch Gladbach, Germany |
| Major Histocompatibility Complex-II (MHC-II) | M5/114,15,2 | eBiosciences | Waltham, MA USA |
| MerTK | DS5MMER | eBiosciences | Waltham, MA USA |
| Programme death protein (PDL) 1 | MIH5 | Becton Dickinson | Franklin Lakes, NJ, USA |
| Transforming Growth Factor (TFG) β | TW7-16B4 | Biolegend | San Diego, CA, USA |
| Tumor necrosis factor (TNF) α | MP6-XT22 | eBiosciences | Waltham, MA USA |
