# Supplementary material for: Improved efficacy and long‐term protective effects of CXCR4/IL10 bioengineered mesenchymal stromal cells in a model of inflammatory bowel disease
Source: Bioeng Transl Med. 2025 Dec 16;11(2):e70083. doi: 10.1002/btm2.70083 (PMC13093540; doi:10.1002/btm2.70083)
Supplement: Supplementary file 9 — TABLE S2: List of primers used for mouse. [file BTM2-11-e70083-s004.docx]

**Supplementary Table 2**. List of primers used for mouse

| **mRNA** | **Forward** | **Reverse** |
| --- | --- | --- |
| Arginase-1 (Arg-1) | CAG AAG AAT GGA AGA GTC AG | CAG ATA TGC AGG GAG TCA CC |
| CD206 | AAC GGA ATG ATT GTG TAG TTC TAG C | TAC AGG ATC AAT AAT TTT TGG CAT T |
| Cyclooxygenase (COX) 2 | GGGAGTCTGGAACATTGTGAA | TGTCAATCAAATATGATCTGGATGT |
| FOXP3 | GGC TGG GAA GAT GGC GCT GG | GCC TCC CGA GGA GCA CA |
| GADPH | ATC AAA TGG GGT GAG GCC GG | TGA CCC TTT TGG CTC CAC CC |
| Inducible Nitric Oxidase Synthase (iNOS) | GAG ACA GGG AAG TCT GAA GCA C | CCA GCA GTA GTT GCT CCT CTT C |
| Interleukin 6 | ACAAAGCCAGAGTCCTTCAGA | TGGTCCTTAGCCACTCCTTC |
| Interleukin 10 | CCC TGG GTG AGA AGC TGA AG | TTC ACC TGC TCC ACT GCC TT |
| Interferon (IFN) γ | TTACTACCTTCTTCAGCAACAGCAA | CTGGTGGACCACTCGGATGA |
| RORC | TGT GCA GGG CCT ACA ATG CC | AAA AAC ACA GGG CGC TGA GG |
| Tbx21 | AAG TGG GTG CAG TGT GGA AA | AAG CCC CCT TGT TGG TG |
| Transforming Growth Factor (TFG) β | TGGAGCAACATGTGGAACT | GTCAGCAGCCGGTTACCA |
| Tumoral Necrosis Factor (TNF) α | CACTTGGTGGTTTGCTACGA | GCCTCCCTCTCATCAGTTCTA |
